# Protein Model and Function Analysis in Quorum-Sensing Pathway of *Vibrio qinghaiensis* sp.-Q67

**DOI:** 10.3390/biology10070638

**Published:** 2021-07-09

**Authors:** Ze-Jun Wang, Fu Chen, Ya-Qian Xu, Peng Huang, Shu-Shen Liu

**Affiliations:** 1 Key Laboratory of Yangtze River Water Environment, Ministry of Education, College of Environmental Science and Engineering, Tongji University, Shanghai 200092, China; 1610387@tongji.edu.cn (Z.-J.W.); 2015yaqian@tongji.edu.cn (Y.-Q.X.); 2 State Key Laboratory of Pollution Control and Resource Reuse, College of Environmental Science and Engineering, Tongji University, Shanghai 200092, China; 1710789@tongji.edu.cn; 3 Department of Environmental Engineering, School of Environmental and Geographical Science, Shanghai Normal University, Shanghai 200234, China; chenfu_jeff@shnu.edu.cn; 4 Shanghai Institute of Pollution Control and Ecological Security, Shanghai 200092, China

**Keywords:** homologous model, autoinducer, luciferase, chimeric protein, double-template modeling, binding pocket

## Abstract

**Simple Summary:**

*Vibrio qinghaiensis* sp.-Q67 (Q67) is a nonpathogenic freshwater luminescent bacterium and has been a focus due to its wide use in the monitoring of environmental pollution and the assessment of toxicity, using the luminescence inhibition rate as the end point. However, the lack of available crystal structures limits the elucidation of the structures of the functional proteins of the quorum-sensing (QS) system that regulates bacterial luminescence in Q67. In this study, homologous models of 19 proteins potentially involved in QS and bioluminescence in Q67 were built using MODELLER. Then, we analyzed the predicted structures and functions of these proteins one by one by combining the existing proteins to explore how the QS pathway of Q67 regulated bioluminescence at the molecular level. This study not only provides a database of predicted protein molecular structures for exploring the mechanisms of the toxicity of chemicals from a molecular point of view, but also indirectly summarizes the research status of crystal structure and function analysis in QS systems, providing references and guidance for follow-up research.

**Abstract:**

Bioluminescent bacteria are mainly found in marine habitats. *Vibrio qinghaiensis* sp.-Q67 (Q67), a nonpathogenic freshwater bacterium, has been a focus due to its wide use in the monitoring of environmental pollution and the assessment of toxicity. However, the lack of available crystal structures limits the elucidation of the structures of the functional proteins of the quorum-sensing (QS) system that regulates bacterial luminescence in Q67. In this study, 19 functional proteins were built through monomer and oligomer modeling based on their coding proteins in the QS system of Q67 using MODELLER. Except for the failure to construct LuxM due to the lack of a suitable template, 18 functional proteins were successfully constructed. Furthermore, the relationships between the function and predicted structures of 19 functional proteins were explored one by one according to the three functional classifications: autoinducer synthases and receptors, signal transmission proteins (phosphotransferases, an RNA chaperone, and a transcriptional regulator), and enzymes involved in bacterial bioluminescence reactions. This is the first analysis of the whole process of bioluminescence regulation from the perspective of nonpathogenic freshwater bacteria at the molecular level. It provides a theoretical basis for the explanation of applications of Q67 in which luminescent inhibition is used as the endpoint.

## 1. Introduction

Bioluminescence is the phenomenon of light emission and production in living organisms, and its biological functions can be categorized into four major groups: defense, counterillumination, prey attraction, and intraspecific communication [1,2,3]. To date, luminous bacteria have been found in five main genera—*Vibrio*, *Photobacterium*, *Aliivibrio*, *Photorhabdus,* and *Shewanella*—belonging to three families: *Vibrionaceae*, *Shewanellaceae,* and *Enterobacteriaceae* [3,4]. Despite their classification into three families, all bioluminescent bacteria share the core sequence *luxCDABE(G)*, which encodes the enzymes necessary for light emission [3]. Luciferase is formed by *α* and *β* subunits encoded by *luxA* and *luxB*, respectively. *luxC*, *luxD,* and *luxE* encode three subunits of the long-chain fatty acid reductase complex needed to synthesize aliphatic aldehyde: reductase, transferase, and synthetase, respectively. Luciferase can catalyze the bioluminescence reaction, whose substrates, aliphatic aldehydes (RCHO) and reduced flavin (FMNH_2_), are synthesized by long-chain fatty acid reductase and flavin reductase. The flavin reductase is encoded by *luxG* in the presence of *luxG* [5]. In the presence of oxygen molecules, RCHO and FMNH_2_ are oxidized to the corresponding acids (RCOOH) and free flavin (FMN), respectively, accompanied by the production of bioluminescence. The bioluminescence reaction can be described as follows [3,6,7]:

$${\mathrm{FMNH}}_{2}+\mathrm{RCHO}+O_{2}\overset{\mathrm{luciferase}}{\to }\mathrm{FMN}+\mathrm{RCOOH}+H_{2}O+\mathrm{hv}$$

**Figure 1:**
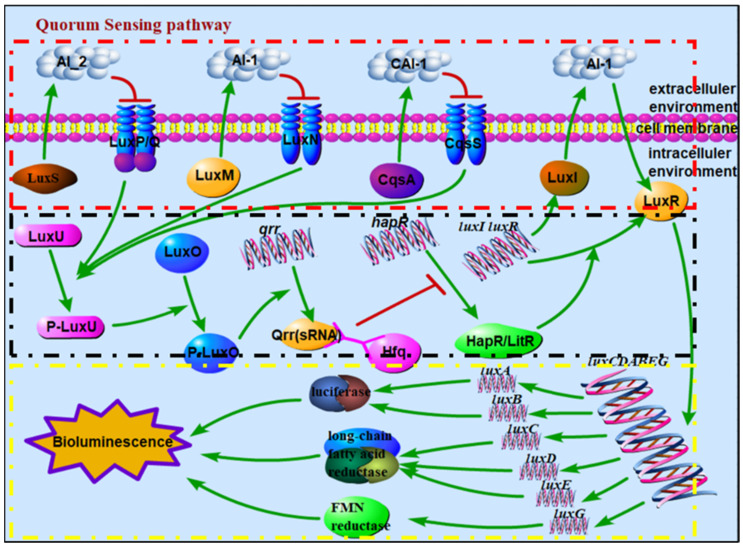
Schematic diagram of signal transmission among related proteins in QS signaling pathway, where the red box refers to autoinducer synthetase sensors, the black box refers to the phosphor-transfer regulation pathway, and the yellow box refers to the mechanism of bioluminescence regulation. (Note: there are LuxS/LuxPQ and CqsA/CqsS in *V. cholerae*; LuxM(AinS)/LuxN(AinR), LuxS/LuxPQ, and CqsA/CqsS in *V. harveyi*; and LuxI/LuxR, LuxM(AinS)/LuxN(AinR), and LuxS/LuxPQ in *A. fischeri*; HapR in *V. cholerae* does not regulate bioluminescence, while LitR in *A. fischeri*. regulates bioluminescence.).

For some luminous bacteria, the bioluminescence is regulated by the quorum-sensing (QS) pathway; the regulatory mechanism in *Aliivibrio fischeri* (*A. fischeri*) and *Vibrio harveyi* (*V. harveyi*) has been investigated in detail [3]. QS is a form of intercellular communication used in bacteria to coordinate group behaviors including virulence, competence, conjugation, antibiotic production, motility, sporulation, and biofilm formation besides bioluminescence [8,9,10]. This process depends on the synthesis and diffusion of signaling molecules, called autoinducers (AIs), into the surrounding environment. Upon reaching threshold concentrations, AIs trigger cellular responses, typically by altering gene expression across the entire population [8,11]. According to the different signal molecules and sensing mechanisms of bacterial biosynthesis, QS systems of *Vibrionaceae* families can be divided into four main types [12,13,14]: LuxI/LuxR, LuxM(AinS)/LuxN(AinR), LuxS/LuxPQ, and CqsA/CqsS (https://www.kegg.jp/kegg-bin/show_pathway?ko02024 (accessed on 1 June 2018). In general, several of these four systems are encoded cooperatively in certain bacteria, such as LuxS/LuxPQ and CqsA/CqsS in *Vibrio cholerae* (*V. cholerae*) [12,15]; LuxM(AinS)/LuxN(AinR), LuxS/LuxPQ, and CqsA/CqsS in *V. harveyi* [13]; and LuxI/LuxR, LuxM(AinS)/LuxN(AinR), and LuxS/LuxPQ in *A. fischeri* [14,16]. In the LuxI/LuxR system, LuxR can form a complex with AI-1, which is synthesized and released by LuxI [17,18], and bind DNA to activate the transcription of the core sequence *luxCDABE(G)* at high bacterial density [19]. When the bacterial density is high enough, the autoinducers (AI-1, AI-2, and CAI-1), which are produced and released by the synthetase proteins LuxM, LuxS, and CqsA, respectively, bind to the corresponding receptors (LuxN, LuxP-LuxQ, and CqsS) [20,21,22]. At this point, the receptor(s) cannot autophosphorylate and inhibit the phosphotransfer step of the regulation pathway. When the regulation pathway is not inhibited, the phosphoryl group is transferred to LuxU and LuxO from the receptor(s), further activating the expression of the Qrr protein, together with the RNA chaperone Hfq, inhibiting the expression of HapR(LitR) [19,23,24,25,26]. Therefore, HapR(LitR) is successfully expressed and activates the expression of the LuxR protein, thereby regulating the bioluminescence. The signal transmission among related proteins in the QS signaling pathway is shown in Figure 1.

In 1985, *Vibrio qinghaiensis* sp.-Q67 (Q67) was firstly isolated and identified from *Gymnocypris przewalskii* in Qinghai Lake of China by Wenjie Zhu, a scholar of East China Normal University [27]. Q67 has been widely used in monitoring environmental pollution and toxicity assessment because it is a nonpathogenic bioluminescent bacterium present in the freshwater ecosystem. Q67 allows the effective detection of the toxicity of pollutants in pesticide residues [28], veterinary drugs [29], and heavy metal pollution [30,31] in a fast, simple and inexpensive manner. In addition, a luminescent bacterial test based on Q67 has been used to assess the ecotoxicity in water samples from wastewater treatment plants (WWTP) [32,33,34]. Furthermore, Q67 is applied to the toxicity detection and assessment of mixtures of pesticides, ionic liquids, antibiotics, wastewater, etc. [35,36,37,38,39,40,41]. It is worth noting that Q67 has also exhibited hormetic phenomena in ionic liquids [42,43], sodium halide salts [44], organic solvent [45], and commercial personal care products [46].

Since the luminescence inhibition rate for the luminescent bacteria is used as the endpoint in toxicity assessment, it is important to study the mechanism of bioluminescence, with the further study of Q67. However, the crystal structure of the protein and substrate complexes in the QS pathway has not yet been determined experimentally, which hinders the study of the related luminescence processes, especially at the molecular level. In this study, we firstly built and refined the monomeric homologous model of 19 proteins in the QS signaling pathway of Q67 using MODELLER; then, we predicted the oligomeric structure based on template proteins or reported functionally similar proteins; and finally, we analyzed the predicted structure-function of the homologous model to explore the binding sites of the proteins and their substrates and the rules of phosphoryl group transmission and other information.

## 2. Materials and Methods

### 2.1. Protein Sequence

The proteins or subunits needed to construct the homologous model include four groups of autoinducer synthetase sensors (LuxI–LuxR, LuxM–LuxN, CqsA–CqsS, and LuxS–LuxPQ), two phosphotransferases (LuxO and LuxU), an RNA chaperone (Hfq), a regulator (HapR(LitR)), and enzymes involved in bacterial bioluminescence: luciferase (LuxAB), long-chain fatty acid reductase (LuxCDE) and flavin reductase (LuxG). The gene sequences of 19 coding sequences were obtained by complete genome sequencing, and the corresponding FASTA sequences were predicted (in Table S1).

### 2.2. Software

The homology or comparative modeling of all the proteins’ three-dimensional structures was constructed, optimized, and evaluated using MODELLER 9.19 [47,48], and the corresponding program was written in Python 3.6. The steps or program for constructing, optimizing, and evaluating the homology or comparative model can be found in the tutorial on MODELLER’s official website (https://salilab.org/modeller/tutorial/, accessed on 10 June 2018). Because templates could not be found for some protein sequences using MODELLER (sequence identity < 30%), the templates of these proteins were searched for using other servers, such as SWISS-MODEL (https://swissmodel.expasy.org/, accessed on 1 September 2018).To evaluate the reliability of the model, third-party software (SAVES v5.0 http://servicesn.mbi.ucla.edu/SAVES/, accessed on 10 November 2018) was used to evaluate the model. In SAVES (v5.0), 4 parameters (Verify3D, ERRAT, Prove, and Ramachandran plot) were chosen to quantitatively characterize the quality of the model. The three-dimensional structures of the protein were visualized in Discovery Studio 2017. Discovery Studio 2017 also provided two-dimensional structure visualization and Ramachandran plots.

### 2.3. Monomeric Protein Modeling

Based on Python 3.6, the homologous model of each protein sequence was built using MODELLER 9.19 according to the following steps: searching for structures related to the target protein from a database of known protein sequence, selecting a template sequentially and structurally, aligning the target protein with the template, model building, loop refining, and model evaluation. The program code for the model building and loop refining, for example, LuxI based on 1ro5_A, is shown in Code S1, and the program codes for the other steps can be found on MODELLER’s official website (https://salilab.org/modeller/tutorial/basic.html, accessed on 10 June 2018). Specifically, for target proteins that could not be completely aligned with templates, homology modeling was performed using appropriate aligned positions of the target protein. Additionally, a model of a chimeric protein was built based on two known structures if necessary. The database of known protein sequences was downloaded on 14 June 2018.

### 2.4. Oligomeric Structure Prediction and Evaluation

Compared to monomeric proteins, the majority of proteins in a living cell exist as parts of complexes and quaternary structure assemblies [49,50]. In addition, ligand binding sites and enzymes’ active sites tend to be located at protein chain interfaces. Therefore, modeling the oligomeric structure of a protein is essential for building models that are useful in biomedical applications [50,51]. Oligomers can be divided into homologous oligomers and heterologous oligomers according to whether the subunits are the same. For both, the subunits must be aligned between target and template proteins one by one; the subunits in a homologous oligomer are the same, while the subunits are different in a heterologous oligomer. It is worth noting that whether to construct oligomers and the numbers of subunits of the oligomers depend on the template proteins or reported functionally similar proteins.

### 2.5. Model Evaluation

The model evaluation consists of two parts: evaluation using MODELLER (9.19) and evaluation using SAVES (v5.0). In MODELLER, the best building model was chosen based on the same template according to the normalized DOPE score [47,52] and GA341score [47,53]. The lower the normalized DOPE score, the better the model, and the higher the GA341 score, the better the model. Moreover, the GA341score can also be used to quantitatively characterize the quality of the model, since it is a nonlinear combination of the protein compactness, combined statistical potential z-score, and percentage sequence identity of the alignment that was used to build the model [53]. The closer the model is to 1, the better its quality is. Empirically, the model was considered high quality is qualified if its GA341 score was more than 0.90. In SAVES (v5.0), 4 parameters (Verify3D, ERRAT, Prove, and Ramachandran plot) were chosen to quantitatively characterize the quality of the model. For Verify3D and ERRAT, the higher the value is, the better the quality of the model is. We set two empirical limits (80.00% for Verify3D and 80.00 for ERRAT) for comparison and analysis. For Prove, a value greater than 5.0% refers to ERROR, one less than 5.0% but greater than 1.0% to WARNING, and one less than 1.0% to PASS. Since Prove is relatively strict, a model with a value less than 5.0% for Prove was considered to pass. For the Ramachandran plot, the confirmation of the model conforms to the stereochemistry rules when the proportion of residues in the core and additionally allowed is higher than 90.0%. Empirically, a model was considered to be reliable if it passed on 2 of 4 parameters. Both monomeric and oligomeric structures were evaluated as above.

## 3. Results and Discussion

Based on the gene sequences of 19 coding sequences obtained by complete genome sequencing, we constructed monomer models of all the proteins or subunits, and the oligomers were predicted based on subunits of template proteins or reported functionally similar proteins for better functional analysis. These proteins or subunits included four groups of autoinducer synthetase sensors (LuxI/LuxR, LuxM/LuxN, CqsA/CqsS, and LuxS/LuxPQ), two phosphotransferases (LuxO and LuxU), an RNA chaperone (Hfq), a regulator (HapR(LitR)), and enzymes involved in bacterial bioluminescence: luciferase (LuxAB), long-chain fatty acid reductase (LuxCDE) and flavin reductase (LuxG).

### 3.1. Homologous Model of Monomers and Oligomers

#### 3.1.1. Modeling of Protein Monomers

The monomer models of 19 gene-coded proteins or subunits were built using MODELLER. Specifically, the chimeric model of CqsS was also constructed because the two regions of CqsS can align with 2c2a and 3luf. Because of the difference in predicted structure between the chimeric model and the monomer model, the chimeric model is called CqsS* for convenience in the study. Table 1 shows 20 target proteins and their template proteins and the predicted three-dimensional structures of the 20 best models are shown in the corresponding figure. Among them, 14 target proteins have reliable templates, while there are 6 proteins (LuxM, LuxN, CqsS, CqsS*, LuxE, and LuxC) that share less than 30% sequence identity with corresponding template sequences (bold in Table 1). It should be noted that not all homologous models are constructed based on the complete sequences because of the incomplete sequences of template proteins. In this study, eight target proteins (LuxP, LuxQ, LuxN, CqsS, CqsS*, LuxO, Hfq, and LuxC) could not be completely aligned with their template protein. For example, the homologous modeling of LuxN, with a length of 859, was built by aligned positions from 450 to 683. The residue alignment and conservation between the target protein and template are shown in Figure S1.

The ribbon diagrams of the proteins and template monomers are shown in Figure S2. It is worth noting that the template protein and homologous model appear to have high similarity by eye. The lower the sequence identity, the more the patternless regions (silvery-white) in the predicted three-dimensional structure, such as for LuxM and LuxE. According to the quantitative evaluation of the models’ quality using MODELLER (in Table 1), the monomeric models of 18 proteins were built very successfully, with GA341 scores higher than 0.90, with LuxM and LuxE being the exceptions. However, in the quantitative evaluation of the models’ quality using SAVES v5.0 (in Table S2), all 20 monomeric models passed according to the Ramachandran plots, while only six monomeric models (CqsA, LuxP, LuxO, HapR(LitR), LuxA and LuxB) passed according to four parameters. The models that passed on three parameters included LuxI, LuxR(VanR), LuxS, Hfq, and LuxD, and another four models (LuxN, LuxQ, LuxU, and LuxG) only passed on two parameters. The five monomeric models (LuxM, CqsS, CqsS*, LuxC, and LuxE) that only passed on one parameter belonged to target proteins with sequence identity lower than 30%. In general, 15 of the 20 monomeric models were reliable.

#### 3.1.2. Modeling of Protein Oligomers

Although the template protein (3qp6) of the monomer model (LuxR) is a monomer, it has been reported that LuxR homologs, such as 3szt, are oligomers. Therefore, oligomer models of LuxR were built based on 3szt rather than 3qp6. Based on template proteins or reported functionally similar proteins, the 12 oligomer models of the target proteins were built using MODELLER. The 12 target proteins and their template proteins are shown in Table 2, and the predicted three-dimensional structures of the 20 best oligomer models are shown in the corresponding figure. The expected common homologous oligomers, two heterologous oligomers (LuxPQ and LuxAB), were successfully constructed. Among them, nine target proteins had reliable templates, while three proteins (LuxN, LuxE, and LuxC) shared less than 30% sequence identity with the corresponding template sequences. Similar to the case for the monomers, the homologous model of the four target proteins (LuxPQ, LuxN, Hfq, and LuxC) was performed using appropriate aligned positions of their target protein.

The ribbon diagrams of the proteins and template oligomers are shown in Figure S3. It is worth noting that the template protein and homologous model appear to be very similar by eye. According to the quantitative evaluation using MODELLER, the oligomeric models of 11 proteins were built very successfully, with GA341 scores being higher 0.90, with LuxE being the exception. However, for the quantitative evaluation according to SAVES v5.0 (in Table S3), all 12 oligomeric models passed based on the Ramachandran plots, while only LuxAB model passed on four parameters. The models that passed on three parameters included CqsA, LuxS, LuxPQ, Hfq, and HapR, and another two models (LuxD and LuxG) only passed on two parameters. The oligomeric models (LuxR(VanR), LuxN, LuxC, and LuxE) only passed on one parameter. In general, eight of the 12 monomeric models were reliable.

In general, 17 functional protein models, including two heterologous oligomers (LuxPQ and LuxAB), were constructed based on the above 19 proteins or subunits. The sequences and topology diagrams and Ramachandran plots of 17 functional protein models are shown in Figures S4 and S5. In particular, CqsS* is considered to be more reliable since the evaluation results for CqsS and CqsS* were similar, while CqsS* contains more residue sequences (subsequent models of CqsS refer to CqsS*). Among the 17 functional protein models, CqsA, LuxS, LuxPQ, Hfq, HapR(LitR), LuxAB, LuxD, and LuxG in oligomer form and LuxI, LuxU, and LuxO in monomer form are considered to be reliable models in aligned positions. For LuxR(VanR) and LuxN, their monomer models are reliable, although the scores of the evaluation parameter ERRAT for their oligomers are slightly lower than the empirical limits. Additionally, LuxC and LuxE in oligomer form and CqsS in monomer form failed the model evaluation because their templates are functionally consistent enzymes rather than complete homologues. Even so, these models have clear characteristic functional domains, which can be used to predict some useful functions. Unfortunately, the homologous modeling of LuxM failed. The relationships between the function and predicted structures of 17 functional proteins were explored one by one according to their functional classification.

### 3.2. Autoinducer Synthases and Receptor

The QS system starts with the synthesis and binding of AIs. For Q67, four groups of gene sequences for synthetases and receptor proteins (LuxI/LuxR, LuxM/LuxN, CqsA/CqsS, and LuxS/LuxPQ) were detected by gene sequencing. Therefore, we first analyzed the function by combining the predicted structures of the four groups of autoinducer synthetase sensors.

#### 3.2.1. LuxI and LuxR

**Figure 2:**
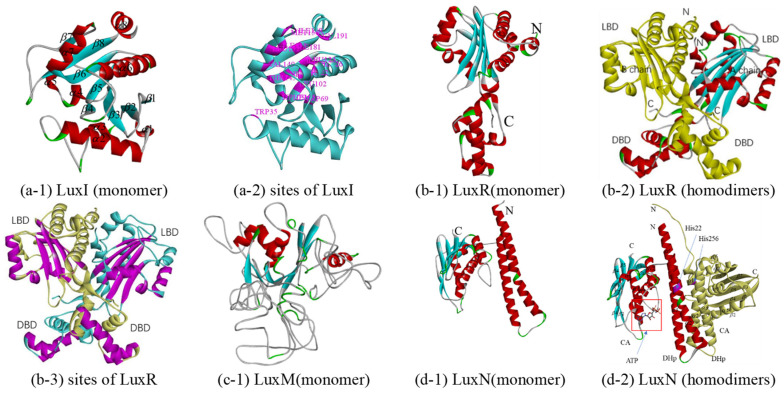
Predicted structure-function diagram of autoinducer synthases and receptor (LuxI/LuxR and LuxM(AinS)/LuxN(AinR)), where the colorful ribbon diagram refers to chain A, yellow to chain B, and “N” and “C” refer to N-terminal and C-terminal of the protein. (**a**-**1**), (**b**-**1**), (**c**-**1**), and (**d**-**1**) are the ribbon diagrams of monomers; (**b**-**2**) and (**d**-**2**) are the ribbon diagram of oligomers, where the red box refers to predicted binding-site and purple refers to the residues of the site; (**a**-**2**) and (**b**-**3**) are the active site or binding pocket site diagram of target proteins.

LuxI is an AHL synthase that produces autoinducer molecules, also known as VAI, such as N-(beta-ketocaproyl) homoserine lactone or 3-oxo-N-(tetrahydro-2-oxo-3-furanyl)-hexanamide, in *A. fischeri*, from the substrates S-adenosyl-L-methionine (SAM) and acyl-acyl-carrier protein (acyl-ACP). However, how acyl-ACP binds to LuxI, which requires a cocrystal structure with acyl-ACP and any AHL-synthase, has not been determined. Although there is no crystal structure of LuxI at present, there are many crystal structures of LuxI homologs [54,55,56], LasI of *Pseudomonas aeruginosa* (PDB Iro5), EsaI of *Erwinia stewartii* (PDB 1kzf and 1k4j), and TofI of *Burkholderia glumae* (PDB 3p2h and 3p2f). Among them, the LuxI from Q67 and Iro5 share the highest sequence identity, 44% (in Table 1). Therefore, the homologous monomer model was constructed based on Iro5. The LuxI structural model is complete from the N-terminal residue Met1 to residue Ala193. The overall predicted structure of LuxI is a three-layer (*αβα*) sandwich containing eight *α*-helices surrounding a highly twisted platform of eight *β*-sheets (in Figure 2(a-1)) and Figure S4a). With reference to LasI, it is predicted that the structure of the acyl-chain binding pocket in LuxI is a tunnel that is formed by the following residues: Trp35, Trp69, Met79, Leu102, Phe105, Thr122, Leu123, Dhe126, Tyr141, Thr145, Val149, Leu152, Ile153, Met156, Ile158, Ile181, and Val191 (purple in Figure 2(a-2)). Based on the structure and residue contacts observed for holo-ACP (ACP with the phosphopantetheine prosthetic group attached to Ser36) binding to acyl-carrier-protein synthase (AcpS; PDB 1F80), the protein position at which ACP interacts with the LasI helix consisting of residues Val146 to Arg154 was predicted [56]. In this way, the helix *α*7 consisting of residues Ala147 to Met156 of LuxI of Q67 is also considered a binding site, which should be determined after analyzing the cocrystal structure with acyl-ACP and any AHL synthase.

**Figure 3:**
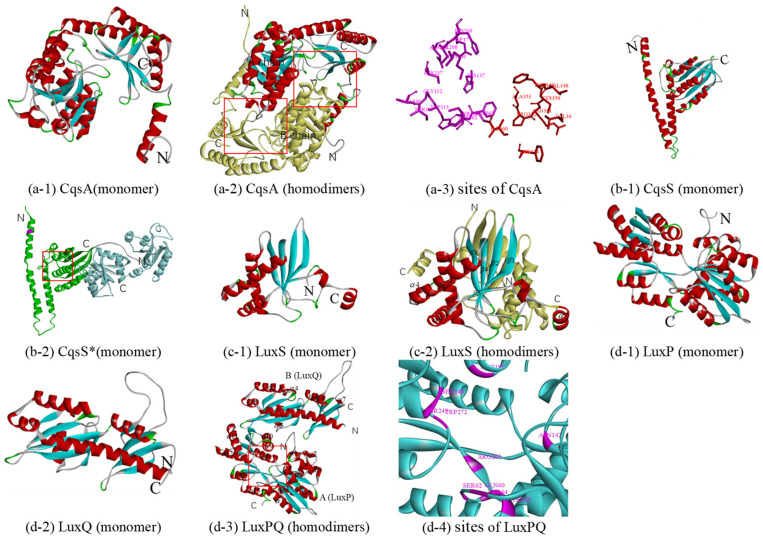
Predicted structure-function diagram for autoinducer synthases and receptor. (**a**-**1**), (**b**-**1**), (**c**-**1**), (**d**-**1**) and (**d**-**2**) are the ribbon diagrams of monomers; (**a**-**2**), (**c**-**2**) and (**d**-**3**) are the ribbon diagram of oligomers, where the red box refers to predicted binding-site and purple refers to the residues of the site; (**a**-**3**) and (**d**-**4**) are the active site or binding pocket site diagram of target proteins. In particular, (**b**-**2**) is a monomer according to double template modeling, where the part templated by 2c2a is colored in green and that of 3luf is blue.

LuxR, free in the cytoplasm, can form a complex with AHL and bind DNA to the activate transcription of QS target genes. LuxR-type proteins fail to fold stably and are therefore degraded by proteases. LuxR-type proteins are homodimers, each monomer of which consists of two domains, a ligand-binding domain (LBD) and a DNA-binding domain (DBD). Although crystal structures of many LuxR family proteins have been reported, few have been crystallized intact [57,58,59,60,61,62], such as LasR of *Pseudomonas aeruginosa* (PDB 6MVN and 6MVM), TraR of *Agrobacterium tumefaciens* (PDB 1H0M, 1L3L, etc.), SdiA (PDB 4y13, 4y15, etc.) of *Escherichia coli*, CviR (PDB 3qp5 and 3qp6) of *Chromobacterium violaceum*, QscR (PDB 3szt, 6cbq and 6cc0) of *Pseudomonas aeruginosa*, and DosR (PDB 3c3w) of *Mycobacterium tuberculosis*. Among them, 3stz could bind to N-3-oxo-dodecanoyl-L-Homoserine Lactone, sharing 30% sequence identity with LuxR of Q67, and was selected as a template for constructing a homologous model (in Table 1 and Figure 2(b-1)). The A-chain contains 15 *α*-helices and five *β*-sheets, while the B chain contains 16 *α*-helices and five *β*-sheets (in Figure 2(b-2) and Figure S4b). The difference between the two subunits is between Leu381 and Ala404, where there are two helices for A but three helices for B. The LBD forms an *α*-*β*-*α* sandwich, and a canonical helix-turn-helix motif forms the DBD. For homodimers, each DBD stacks underneath the LBD from the same monomer. However, in some cases, such as binding with antagonists, the dimer structure changes so that it cannot bind to DNA. For example, in the antagonist-bound CviR structure, the DBD of each monomer is positioned below the LBD of the opposite monomer [59]. For LuxR of Q67, it is predicted that the AHL binds in a pocket formed between the *α*-helices *α*3–*α*5 and *β*-sheets *β*1–*β*5, and the helices *α*13 and *α*14 of A chain and *α*14 and *α*15 of B chain in DBD are predicted to be required for binding to DNA (purple in Figure 2(b-3)).

#### 3.2.2. LuxM and LuxN

Similar to LuxI, LuxM is also an AHL synthases that synthesizes N-acyl-L-homoserine lactone through SAM and acyl-ACP [63]. At present, there are no reports on the crystal structures of LuxM. The sequence identity between LuxI and LuxM is very low, although their functions are similar. These two issues limit the elucidation of the predicted structure of a functional LuxM protein. There is no suitable template in MODELLER, and a poor template, 3p2h (10% sequence identity), was selected by Swiss-model (Table 1). 3p2h is the acyl-homoserine lactone synthase encoded by the *tofi* gene of *Burkholderia glumae*, which is, in essence, still a LuxI homologue. The homology modeling of LuxM failed because the involvement of individual amino acids and specific structural elements could not be determined from the model, which is due to a large number of loop areas in the model with low sequence identity (in Figure 2(c-1)).

LuxN is also a membrane-bound hybrid sensor kinase protein, and it can respond to autoinducer-1 released by LuxM and autophosphorylates, thereby a transmitting phosphoryl group to LuxU [21]. Since no structural information of LuxN has yet been reported, homology models were attempted to be generated based on sensor histidine kinase. Sensor histidine kinase WalK from *Lactobacillus plantarum* shares a higher sequence identity, 26%, with LuxN from Q67 and PDB 4u7o, compared to the two other crystal structures (PDB 4u7n and 4zki), which have the highest resolution of 2.4Å (in Table 1). Each monomer of the homologous model consists of two domains: a dimerization and histidine phosphorylation domain (DHp) and a catalytic ATP-binding domain (CA); two monomers compose dimers consisting of DHp (monomer in Figure 2(d-1)) and homodimers in Figure 2d-2). Referring to kinase activity of LuxN from *V. harveyi* [21] and WalK from *Lactobacillus plantarum* [64], the His22 and His256 of the LuxN of Q67 (purple in Figure 2(d-2)) are required for kinase activity (but not for phosphatase activity) and can autophosphorylate in the absence of AI-1. The predicted pocket bound to ATP is formed by the *α*-helices *α*4,*α*5, and *α*7. However, in the case of AI, the knowledge of the LuxN-AI-1 complex is incomplete due to the limited sequence of crystal structure information for the LuxN.

#### 3.2.3. CqsA and CqsS

It is generally believed that CqsA is required for the synthesis of the (S)-3-hydroxytridecan-4-one (CAI-1). In 2009, based on the acyl-CoA transferase 8-amino-7-oxononanoate synthase (AONS) reaction, a possible synthetic pathway using (S)-2-aminobutyrate (SAB) and decanoyl-coenzyme A (dCoA) as substrates was proposed based on the pyridoxal phosphate (PLP)-dependent acyl-CoA transferase reaction [65]. In the reaction, amino-CAI-1 was produced by CqsA and then converted to CAI-1 via a CqsA-independent mechanism. At the same time, the existence of a similar reaction was confirmed by crystal structure and mass spectrometry analysis [66]. To date, although all six reported crystal structures of CqsA belong to *V. cholerae* (PDB 2wk7, 2wk8, 2wk9, 2wka, 3hqt, and 3kki), no structural data have been obtained on CqsA with substrates such as dCOA, while crystal structures of CqsA with PLP have been reported because CqsA is a PLP-dependent enzyme [66]. Since these crystal structures share a high similarity and sequence identity (around 68%) with the CqsA of Q67, 3kki, with the highest crystal resolution (1.8 Å), was chosen as the template (Table 1 and monomer in Figure 3(a-1)). Although the rotated symmetric A and B chains have the same helix number (18) and same sheet number (12), there are differences between them: (1) 12–25 residues form two helices (*α*1 and *α*2) in the A chain, while the corresponding position only forms one helix (*α*1) in the B chain; and (2) 480–482 residues form a helix (*α*4) in the B chain (in Figure S4e-1). Each monomer of a CqsA can be described as containing three domains, all of which are involved in dimer formation: an N-terminal domain (a helix followed by the random coil), a central catalytic domain (around residues 57–286), and a C-terminal domain (in Figure 3(a-2)). In the dimer CqsA of Q67, there are two pockets (Figure 3(a-2)), one of which is formed by residues Ser111, Gly112, Trp113, His137, Asp177, Asp206, Ser208, His209, Ser237, Lys240, Ala246, Phe661, Ser662, and Ser663 (purple in Figure 3(a-3)), which refers to key residues surrounding the PLP cofactor in the 3kki. Additionally, two pockets of CqsA in Q67, near to two PLP-binding pockets formed by Val36, Val342, Phe349, Cys350, Arg351, Pro352, Ala353, Phe654, and Ile660, could bind the substrate 2,3-dihydroxy-1,4-dithiobutane (red in Figure 3(a-3)), which also refers to 3kki, although the function of the substrate has not been reported.

Like LuxN, in the absence of CAI-1, the inner membrane sensor kinase CqsS functions as a kinase and autophosphorylates on a histidine residue, while CqsS bound to CAI-1 functions as a phosphatase rather than kinase in the presence of CAI-1, inhibiting the phosphotransfer pathway. However, how CAI-1 binds to CqsS has not been determined, since there is no crystal structure of CqsS at present. After sequence alignment, it was found that the two structures of the kinase represented by 2c2a and 3luf corresponded to different positions of protein sequences. 2c2a is the crystal structure of sensor histidine kinase (positions 232 to 489 of 489 residues) from *Thermotoga maritima*. 3luf is the crystal structure of two-component system response regulator/GGDEF domain protein (positions 2 to 249 of 415 residues) from *Aeromonas salmonicida*. Therefore, a possible protein structure of the CqsS monomer (positions 180 to 683 of 683 residues) was constructed using double template modeling; the 180–413 region was based on the template 2c2a (25% sequence identity), and the 428–683 region was based on the template 3luf (21% sequence identity) (Table 1 and Figure S2, CqsS*). The predicted structure templated by 2c2a (green in Figure 3(b-2)) consists of two distinct domains, an N-terminal helical hairpin domain and a C-terminal *α*/*β* domain, which are connected by a short linker, and it is similar to the corresponding domains of other kinases, such as *Thermotoga maritima* (PDB 2c2a, 3a0s, etc.); *Lactobacillus plantarum* (4u7n, 4u7o, and 4zki), and *E. coli* (PDB 4biu, 4biv, etc.). The predicted structure templated by 3luf consists of two similar three-layer (*α–β–α*) sandwiches (blue in Figure 3(b-2)), and it is similar to the corresponding domains of RcsC of *E.coli* (PDB 2ayx) and PleD of *Caulobacter vibrioides* (PDB 1w25, 2wb4, and 2v0n). There are 13 histidine residues in CqsS* of Q67, six in the domain templated by 2c2a, and seven in the domain templated by 3luf. Only one histidine residue exists in the DHp domain (purple in Figure 3(b-2)), and it may autophosphorylate in the absence of CAI-1, while for the binding pocket of CAI-1, it is in the conserved position of the binding pocket of ADP (red box in Figure 3(b-2)) and remains to be further studied.

#### 3.2.4. LuxS and LuxPQ

LuxS, a Zn^2+^-dependent metalloenzyme, is involved in the synthesis of autoinducer -2 (AI-2), regulating gene expression in some QS systems such as in certain *Vibrio* species and some *E. coli* strains. It can catalyze the transformation of S-ribosylhomocysteine (RHC) to homocysteine (HC) and 4,5-dihydroxy-2,3-pentadione (DPD), which is the direct precursor of AI-2, with multiple intermediates formed. The crystal structures of many LuxS proteins have been reported [67,68], such as LuxS (PDB 1ie0, 1j98, etc.) of *Bacillus subtilis*, LuxS (PDB 1j6W and 1joE) of *Haemophilus influenza*, LuxS (PDB 1inN, 1j6V, etc.) of *Deinococcus radiodurans*, LuxS (PDB 5e68 and 5v2W) of *Salmonella typhi*, LuxS (PDB 1j6X) of *Helicobacter pylori*, and LuxS (PDB 4xcH) of *Streptococcus suis*. Among them, 5e68 was selected as a template for constructing a homologous model due to its 72% sequence identity with the LuxS of Q67 (in Table 1). The LuxS monomer (Figure 3(c-1)) consists of a four-stranded antiparallel sheet and a vertical sheet in contact with seven helices, while in the homodimer (in Figure 3(c-2) and Figure S4), the A and B chains are almost rotated symmetrically except helices *α*7. According to studies of LuxS in other organisms, identical active sites, which can be bound by metals and substrates, are formed at the dimer interface in the LuxS of Q67. For example, two metal-binding sites [67] may be formed by His 54, His58, and Cys128 in the A or B chain in the LuxS of Q67. The pocket of the RHC [69], intermediates [70], and HC [69], near metal-binding sites, is formed between Cys300 (around), helices *α*1, and helices *α*3 with Leu3 (around) and *β*5 of another chain in the LuxS of Q67.

**Figure 4:**
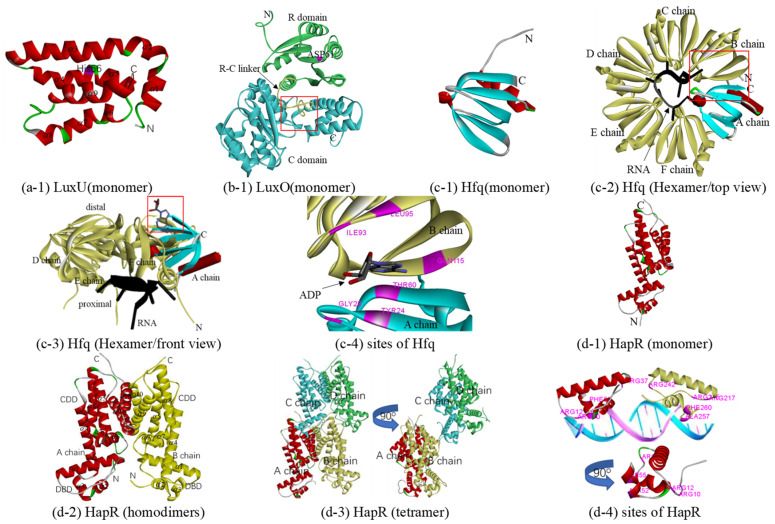
Predicted structure-function diagram of signal transmission protein: phosphotransferase, RNA chaperone, and transcriptional regulator. (**a**-**1**), (**b**-**1**), (**c**-**1**), (**d**-**1**) and (**d**-**2**) are the ribbon diagrams of monomers, where the red box refers to the predicted binding-site and purple refers to the residues of the site; (**c**-**2**) and (**c**-**3**) are a top and front view of the Hfq-RNA complex, respectively, and (**c**-**4**) shows the binding sites of ADP; (**d**-**2**) and (**d**-**3**) are homodimers and tetramer of HapR, and (**d**-**4**) is basic residues for binding DNA (Arg10, Arg12, Arg37, Ala52, and Phe55 of A chain and Arg215, Arg217, Arg242, Ala257, and Phe260 of B chain).

LuxPQ is a receptor of the autoinducer AI-2 released by the synthetase protein of LuxS. At low cell density, in the absence of AI-2, the inner membrane sensor kinase LuxQ functions as a kinase and autophosphorylates on a histidine residue; then, the phosphoryl group is transferred to LuxU and, ultimately, to LuxO. By contrast, at high cell density, in the presence of AI-2, the binding of AI-2 to the receptor LuxP, a periplasmic binding protein, modulates the activity of LuxQ, making it function as a phosphatase rather than kinase, transducing the AI-2 information into the cytoplasm. The first crystal structure of the AI-2 receptor was the complex LuxP-AI-2 from *V. harveyi* (PDB 1jx6) [71], which determined the binding pocket of AI-2. Then, in 2005, it was proved that LuxP and LuxQ_p_ form heterodimers in both the presence and absence of AI-2 (PDB 1zhh) [22]. Furthermore, crystal structures of the isolated periplasmic domain of LuxQ (LuxQ_p_) and LuxPQ_p_ bound to AI-2 (tetramer) were present (PDB 2HJ9) [72]. Additionally, there are other crystal structures of LuxP or LuxQ, such as LuxP (PDB 4yp9, 4yr7, and 4yrz) and LuxQ (PDB 2hje) of *V. harveyi* and LuxQ (PDB 3c38 and 3c30) of *V. cholerae*. Considering the sequence identity and resolution, the best template is 1zhh, with a resolution of 1.94 Å, two chains of which and LuxPQ of Q67 show 65% and 37% sequence identity, respectively. LuxP, containing 15 *α*-helices and 12 *β*-sheets, consists of two similar domains connected by a three-stranded hinge (Figure 3(d-1)), while LuxQ, containing seven *α*-helices and nine *β*-sheets, is composed of two tandem PAS folds (Figure 3(d-2) and Figure S4h). It is worth noting that LuxQ, as a histidine kinase, should also include the typical DHp and CA domains of the kinase; the reason for their absence is incomplete alignment caused by the lack of crystallization in these domains of the template protein (Table 1). LuxP and LuxQ form heterodimers (Figure 3(d-3)). In the absence of AI-2, the phosphoryl group was from the autophosphorylated histidine (His) to aspartate (Asp) residues; however, it is not clear which of the four His (H) and 17 Asp (D) residues are involved in Q67 (Figure S4h). In the presence of AI-2 in Q67, AI-2 binds to a pocket formed by follow residues (purple in Figure 3(d-4))—Gln60, Ser62, Tyr64, Trp65, Asn142, Arg198, Ser248, Thr249, Trp272, and Arg292—which refers to 1zhh, and the phosphotransfer pathway is inhibited.

### 3.3. Signal Transmission Protein: Phosphotransferase, RNA Chaperone, and Transcriptional Regulator

For the LuxI/LuxR system, bacterial luminescence is directly regulated by LuxR. However, the remaining three groups of autoinducer synthetase sensors regulate LuxR to regulate bacterial luminescence through a series of signal transduction processes, involving two phosphotransferases, an RNA chaperone, and a transcriptional regulator.

#### 3.3.1. Phosphotransferase: LuxU and LuxO

As a phosphotransferase, LuxU receives sensory signals, a phosphoryl group, from LuxN, LuxQ, and CqsS and transmits them to LuxO, at low cell density. At high cell density, LuxU can transfer a phosphate from LuxO to LuxN (probably LuxQ and CqsS), and phosphate is ultimately depleted from the system [23,73]. At present, the only crystal structure of LuxU is 1y6d from *V. harveyi*, proposed in 2005 [74], which includes 19 models with little difference from each other. Model 1 of 1y6d with 50% sequence identity was selected as a template for constructing a homologous model of LuxU from Q67 (in Table 1). The overall predicted structure is that of a four antiparallel helix-bundle twisted about the central axis, which consists of nine *α*-helices (in Figure 4(a-1)). It is a member of the histidine phosphotransfer (HPt) proteins; there are three histidine residues in the LuxU of Q67 (Figure S4i), among which histidine residue 56 (His56) is the most likely site of phosphorylation (Figure 4(a-1)).

At low cell density, after receiving phosphoryl groups from LuxU, phosphorylated LuxO activates the transcription of the genes encoding the small RNAs (sRNAs) Qrr, which, together with the protein Hfq, post-transcriptionally repress HapR(LitR), while HapR(LitR) production is derepressed and inhibited, since dephosphorylated LuxO cannot activate the transcription of Qrr at high cell density [24,75,76]. Belonging to the subfamily of AAA+ ATPases known as bacterial enhancer-binding proteins (bEBPs), each monomer of LuxO contains three domains: an N-terminal receiver (R) domain, a central ATPase (C) domain, and a C-terminal sequence-specific DNA-binding (D) domain. In the case of the phosphorylation of an aspartate in the R domain, ATP hydrolysis and the opening/activation of σ54-dependent promoters, which is required for an activator, are activated in the C domain. After that, LuxO activates the transcription of Qrr in D domains [25,77]. At present, although the crystal structure of LuxO with the D domain has not been elucidated, there are several crystal structures of LuxO with R or/and C domain(s) including LuxO with R and C domains (PDB 5EP0) and LuxO with the C domain (PDB 5EP1, 5EP2, 5EP3, and 5EP4) of *Photobacterium angustum* and LuxO with the R domain of *Vibrio parahaemolyticus serotype O3:K6* (PDB 3CFY). In this study, a predicted protein structure of a LuxO monomer with the R and C domains was constructed based on the template 5EP0 (73% sequence identity). Containing six *α*-helices and five *β*-strands in the N terminal, the R domain is nestled between the two lobes of the C domain, one of which consists of five *α*-helices and two *β*-strands in the C terminal, and the other comprises ten *α*-helices and nine *β*-strands (Figure 4(b-1)) and Figure S4, LuxO). The R and C domains are connected by a 19-residue linker (denoted the R-C linker, residues Ala129–Phe147). The predicted phosphorylation site is ASP61 in the R domain. The predicted ATPase active site in the LuxO of Q67 is formed by the *α*-helices *α*7, *α*8, *α*17, *α*18, and *α*20 (Figure 4(b-1)). However, like 5EP0 [25], the R-C linker in the LuxO of Q67 occupies a substantial portion of the ATPase active site (red box in Figure 4(b-1)), which might be capable of inhibiting the ATPase activity. After phosphorylation, the R-C linker is dislocated from the ATP binding site to free the ATPase active site for catalysis.

#### 3.3.2. RNA Chaperone: Hfq

Hfq, an RNA chaperone, can bind small regulatory RNA (sRNAs) and mRNAs to regulate the translation of mRNAs in response to envelope stress, environmental stress, and changes in metabolite concentrations. In the QS system, Hfq, together with Qrr, mainly affects the translation of the mRNA for *hapR* [78], to a certain extent, as well as *luxM* and *luxO* [79]. They also regulate the translation of vca0939, which encodes a diguanylate cyclase that controls biofilm formation in *V. cholerae* [80,81]. Hfq includes six similar monomers, each of which consists of a flexible C-terminal tail and a conserved N-terminal Sm domain. Many crystal structures have been reported to date, most of which contain conserved N-terminal Sm domain with a C-terminal tail, such as Hfq from *E. coli* (PDB 3rer, 3res, etc.) [82] and *Salmonella typhimurium* (PDB 2ylb) [83]. Because 3rer, the RNA-binding protein Hfq from *E. coli*, with a resolution of 1.94 Å, shares the highest sequence identity, 95% (in Table 1), it was considered the best template to construct a homologous model of the Sm domain from the Hfq of Q67. Each monomer of Hfq adopts the Sm fold of an N-terminal *α*-helix followed by five *β*-strands with an interrupted *α*-helix (Figure 4(c-1)). Six similar monomers form a ring from which the disordered C-terminal regions extend outward to produce an overall star-shaped structure (Figure 4(c-2)). One side of the ring is named the proximal side, which preferentially binds most sRNAs and their mRNA targets (Figure 4(c-3)); for example, binding sites of ADP were predicted and were shown in Figure 4(c-4). The opposite side is called the distal side, which is specific for poly(A) or A–R–N (where R is a purine, and N is any nucleotide) triplets [78]. Although there is no crystal structure for the complex of Hfq with Qrr at present, in 2012, Vincent et al. successfully constructed a Hfq-Qrr complex model [78], which could catalytically repress the quorum-sensing regulators *V. harveyi* LuxR [79,84] and *A. fischeri* LitR or *V. cholerae* HapR [19]. In *V. cholerae*, the Hfq-Qrr complex model showed that a ring-shaped Hfq hexamer binds the Qrrs with 1:1 stoichiometry through its proximal face, which may also elucidate the interactions involved between Hfq and the Qrr sRNAs of Q67 (Figure 4(c-2,c-3)).

#### 3.3.3. Transcriptional Regulator: HapR(LitR)

In the QS circuit, there is a type of LitR homolog that acts as a transcriptional regulator, including HapR in *V. cholerae*, LitR in *A. fischeri*, SmcR in *Vibrio vulnificus*, OpaR in *Vibrio parahaemolyticus*, VanT in *Vibrio anguillarum,* and VtpR in *Vibrio tubiashii*. Under high cell density, these transcribed regulator proteins regulate many important cellular processes via QS. HapR represses both virulence gene expression [85] and biofilm formation in *V. cholerae* [86]; LitR enhances LuxR expression, thereby contributing to light production in *A. fischeri* [26]; in Q67, HapR(LitR) may be the transcriptional regulator that enhances LuxR expression for bioluminescence. To date, crystal structures of HapR from *V. cholerae* (PDB 2pbx, 5l0x, and 6d7y) and SmcR in *Vibrio vulnificus* (PDB 3kz9 5 × 3r) have been reported. The 3kz9, with 81% sequence identity, was selected as a template for constructing a homologous model of HapR(LitR) from Q67, including a monomer on the A chain of 3kz9 (Table 1 and Figure 4(d-1)), homodimers on the AB chain of 3kz9 (Figure 4(d-2)), and a tetramer on the ABCD chain of 3kz9 (Table 2 and Figure 4(d-3)). Each monomer contains ten *α*-helices, the first three of which form a DNA-binding domain (DBD), and the remaining seven form a C-terminal dimerization domain (CDD) (Figure 4(d-2)) and Figure S4 HapR). Two chains of HapR(LitR) in Q67 in the homodimer are related by a 2-fold rotation axis (Figure 4(d-2)), and the tetramer consisted of two homodimers (Figure 4(d-3)). Basic residues, including Arg10, Arg12, Arg37, Ala52, and Phe55 of the A chain and Arg215, Arg217, Arg242, Ala257, and Phe260 of the B chain, predicted to bind with DNA sugar-phosphate backbones, are displayed in Figure 4(d-4)).

### 3.4. Enzymes in the Bacterial Bioluminescence Reaction

Bacterial bioluminescence is produced by a bioluminescence reaction catalyzed by enzymes encoded by the core *lux* operon *luxCDABEG*. Luciferase (LuxAB) can catalyze the bioluminescence reaction, whose substrates, aliphatic aldehydes (RCHO) and reduced flavin (FMNH_2_), are synthesized by long-chain fatty acid reductase (LuxCDE) and flavin reductase (LuxG).

#### 3.4.1. Luciferase: LuxA and LuxB

**Figure 5:**
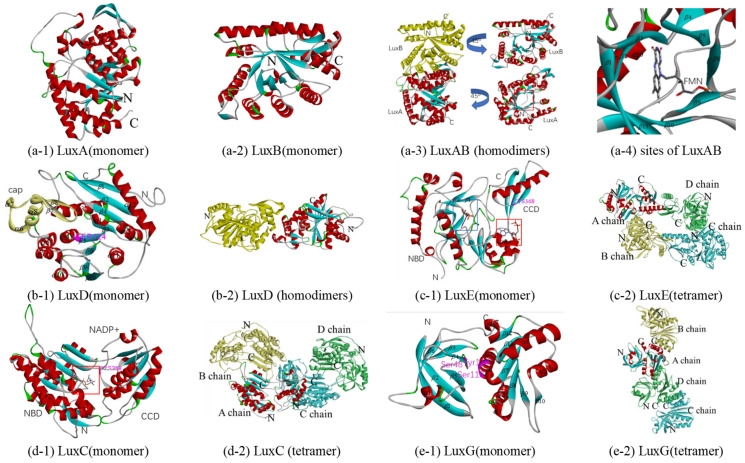
Predicted structure-function diagram of enzymes or their subunits in bacterial bioluminescence. (**a**-**1**), (**a**-**2**), (**b**-**1**), (**c**-**1**), (**d**-**1**) and (**e**-**1**) are the ribbon diagrams of monomers; (**a**-**3**), (**b**-**2**), (**c**-**2**), (**d**-**2**) and (**e**-**2**) are the ribbon diagram of oligomers, where red box refers to predicted binding-site and purple refers to the residues of the site; (**a**-**4**) is the possible conformations of FMN and its surrounding residues.

In bioluminescent bacteria, the heterodimeric enzyme luciferase, the *α*- and *β*-subunits of which are LuxA and LuxB, catalyzes the conversion of aliphatic aldehydes and FMNH_2_ to the corresponding acids and FMN with luminescence, respectively. The reported crystal structures of bacterial luciferase are mainly concentrated in *V. harveyi* (PDB 1brl, 1bsl, 1luc, 1xkj, and 3fgc) [87,88,89,90]. The crystal structures of LuxB from *Photobacterium leiognathid* (PDB 1nfp and 6fri), and bacterial luciferase family proteins (PDB 1rhc, 1z69 and 5lxe) have also been reported [89,91,92,93]. Homologous models of the luciferase of Q67 were constructed based on 3fgc, with a resolution of 2.3 Å, two chains of which and the luciferase of Q67 have 84% (A) and 61% (B) sequence identity, respectively (Table 1 and Table 2). LuxA, the *α*-subunit of luciferase, consists of 16 *α*-helices surrounding a barrel of 13 *β*-strands, and LuxB, the *β*-subunit, consists of 12 *α*-helices surrounding a barrel of 11 *β*-strands (Figure 5(a-1,a-2) and Figure S5 LuxAB). Like the luciferase of *V. harveyi* [87,90,94], luciferase of Q67 is also composed of two homologous subunits, *α* and *β*, both of which folds into a single domain (*β*/*α*)_8_ barrel motif (Figure 5(a-3)). It is generally believed that the active site enabling light emission is located in the *α*-subunit, while the *β*-subunit stabilizes the active conformation of the *α*-subunit, thereby participating in the catalytic mechanism [3,90]. The crystal structure of FMN, the product of the reaction, bound to *V. harveyi* luciferase has been reported, while the crystals of the luciferase/FMNH_2_ complex degraded rapidly and were unsuitable for data collection [90]. In Q67, FMN may bind to the active site pocket formed by eight *β*-sheets (*β*1, *β*2, *β*3, *β*5, *β*8, *β9*, *β*10, and *β*13) in the *α*-subunit (Figure 5(a-3,a-4)), and there is a similar pocket in the *β*-subunit (Figure 5(a-3)), which may bind other small molecules to affect the activity of the whole enzyme, such as hermetic organic solvents [45]. However, no structural information on the spatial orientation of the luciferase with the substrate (FMNH_2_ and the aldehyde) or intermediates of the reaction is available, preventing a more detailed interpretation of how the overall reaction is generated.

#### 3.4.2. Fatty Acid Reductase Complex: LuxC, LuxD, and LuxE

In the bioluminescence reaction, transferase LuxD (encoded by *luxD*), a synthetase LuxE (encoded by *luxE*), and a reductase LuxC (encoded by *luxC*) are required to produce the long-chain fatty aldehyde (*RCHO*) substrates. Although the LuxC_4_LuxD_4_LuxE_4_ complex model based on the homologous models in combination with available functional data and the conservation of residues among LuxCDE-containing organisms has been proposed [3], there are no reports about the crystal structure of the LuxCDE complex at present. Therefore, the independent homologous models were constructed with appropriate templates.

Initially, the transferase (encoded by *luxD*) takes over the acyl moiety from acyl-ACP or acyl-CoA using the alcohol group of a serine residue to form an ester derivative, which is eventually hydrolyzed to the free acid [3,95]. The itht (PDB ID) is the unique crystal structure of LuxD reported at present, which is from *V. harveyi* [96]. Sharing 69% sequence identity with the LuxD of Q67, itht, with 2.1Å resolution, was selected as a template for constructing a homologous model (Table 1). The monomer consists of two domains, a ‘cap’ dimerization domain with 3 *α*-helices (*α*6, *α*7, and *α*8) and two *β*-strands (*β*7 and *β*8), and the main domain with a three-layer (*α*-*β*-*α*) sandwich of nine *α*-helices and eight *β*-strands (Figure 5(b-1)). In the main domain of LuxD in Q67, the acyl could bind to Ser124, between *β*5 and *α* 4, which can form an ester derivative (RCOO-Ser124 (LuxD)), thereby hydrolyzing to the free acid (RCOOH). The dimer is formed through the ‘cap’ dimerization domain by two similar monomers (Figure 5(b-2)).

The free acid (RCOOH) is activated to acyl-AMP by the synthetase LuxE at the expense of ATP; then it can bind to the cysteine residue to form the corresponding thioester (RCOS-Cys(LuxE)) of LuxE, with the release of AMP [3,97,98]. Since there is no structural information on LuxE reported at present, homologous models are attempted to be generated based on acyl-CoA synthetases. The acyl-CoA synthetase of *Bacteroides thetaiotaomicron* (PDB 4rvn), with 2.1Å resolution, and the LuxE of Q67 share the highest sequence identity, 19% (Table 1). The monomer consists of two domains, a C-terminal catalytic domain (CCD) with one *α*-helix (*α*10) and three *β*-strands (*β*14, *β*15, and *β*16) and an N-terminal binding domain (NBD), which could bind CoA and AMP (Figure 5(c-1)). Since Cys364 in the C-terminal is the acyl transfer site of the fatty acyl-protein synthetase in *P. phosphoreum* [97], Cys368 in CCD is predicted to be the site of acyl transfer on the LuxE from Q67, and the corresponding acylated intermediate is RCOS-Cys368(LuxE). For LuxE from Q67, the cavity in NBD composed of *β*1, *β*2, and other residues between *β*6 and *β*7 is predicted as a pocket bound to AMP, which is shown by the blue box, while CoA could bind to *α*8 in NBD in the red box (Figure 5(c-1)). Four similar subunits form the tetramer (Figure 5(c-2)) by a rotation axis, and each subunit contains an acyl transfer site and binding pockets for CoA and AMP.

The fatty acyl group is transferred from LuxE (RCOS-Cys (LuxE)) onto LuxC forming a covalent acylated intermediate ((RCOS-Cys (LuxC))) before being reduced with NADPH to aldehyde (RCOOH) [3,98]. Similar to LuxE, it was attempted to generate homologous models based on aldehyde reductase, since no structural information on LuxC has yet been reported. Retinal dehydrogenase 1A1 from *Ovis aries* (PDB 1bxs, 5abm, 5ac0, and 5ac1) and LuxE of Q67 show the highest sequence identity, 16% (Table 1). Among them, 5abm, with 1.7Å resolution (the highest), was selected as a template for constructing a homologous model (Table 1 and Table 2). The monomer is made up of two domains: an N-terminal NADP^+^-binding domain (NBD) formed by a three-layer (*αβα*) sandwich of twelve *α*-helices and nine *β*-strands and a C-terminal catalytic domain (CCD) consisting of a three-layer (*α*-*β*-*α*) sandwich of six *α*-helices and five *β*-strands (Figure 5(d-1)). The pocket bound to NADP^+^ is located in the red box, between NBD and CCD (Figure 5(d-1)). Since Cys286, located in the segment sequence ‘YDQQACFSTQ’, is the site of the acylation of the Lux-specific fatty acyl-CoA reductase in *P. phosphoreum* [98], Cys289 in CCD is predicted to be the site of acylation on the LuxC from Q67, and the corresponding acylated intermediate is RCOS-Cys289(LuxC). The NBDs of two chains in the homodimer are related by a 2-fold rotation axis and the tetramer consisted of two homodimers (Figure 5(d-2)).

#### 3.4.3. Flavin Reductase: LuxG

In 2008, the function of LuxG was determined as an NAD(P)H-dependent flavin reductase [5]. Free flavin (FMN) could be converted to reduced flavin (FMNH_2_) involved in bacterial bioluminescence, by LuxG. Although the LuxG–LuxAB complex may be formed to directly transfer reduced FMN from the reductase to the luciferase, attempts to establish the formation of such a reductase–luciferase complex have been unsuccessful to date [3]. Although there is no report on the crystal structure of bacterial LuxG, the crystal structures of NAD(P)H-dependent flavin reductases, such as Fre [99], have been studied in detail. Among them, the NAD(P)H-flavin reductase Fre from *E. coli* (PDB 1qfj), with a 2.2Å resolution, and LuxG from Q67 show the highest sequence identity, 36% (Table 1). Therefore, the 1qfj was selected as a template for constructing a homologous model of LuxG from Q67, and the mechanistic details including the substrate specificity and NAD(P)H binding can be inferred from the homologous model. The monomer consists of two domains, an N-terminal domain and a C-terminal domain (Figure 5(e-1)). The N-terminal domain is formed by one *α*-helix and six *β*-strands, while the C-terminal domain is a three-layer (*α*-*β*-*α*) sandwich of six *α*-helices and six *β*-strands (Figure 5(e-1)). A tetramer was formed by four similar monomers in the asymmetric unit shown in Figure 5(e-2). Knowledge of the substrates reduced riboflavin and NAD(P)H bound to LuxG could be inferred from the flavin reductase Fre [99]. In Q67, reduced riboflavin could bind to the isoalloxazine pocket formed by Ser48, Ser118, and Tyr119, and the pocket bound to NAD(P)H comprised *α*-helices *α*2, *α*3, and *α*4 (Figure 5(e-1)).

### 3.5. Homologous Model and Its Application in Q67

The sequence and three-dimensional structure of a protein determine its function. However, the experimental structural characterization of protein sequences by X-ray crystallography and nuclear magnetic resonance (NMR) spectroscopy is limited by the inherent cost, time, and experimental challenges [52]. In the absence of an experimentally determined structure, comparative or homology modeling can often provide a useful 3-D model for a protein that is related to at least one known protein structure [47]. Homology modeling can also be used to explore the structures of unknown molecules. Brodl et al. analyzed the evolutionary conservation of residues at the LuxC and LuxE molecular interface based on the homologous model constructed and predicted tentative model (LuxC4LuxE4 and LuxC4LuxD4LuxE4 complex) combined with the crystal structure of LuxD, thereby revealing the mechanism of luminescence in bacteria [3]. In this study, in addition to building 19 models based on a single template, a novel structure, a chimeric model of CqsS (CqsS*), is proposed based on the double template. Although the two templates (2c2a and 3luf) have a low sequence identity, the evaluation results show that the homologous model is reliable. Regarding function, the two template proteins are a sensor histidine kinase from *Thermotoga maritima* and a two-component system response regulator/GGDEF domain protein from *Aeromonas salmonicida*, respectively. For LuxPQ, with a similar function, the periplasmic binding protein LuxP could bind AI-2 to make the inner membrane sensor kinase LuxQ act as a phosphatase rather than kinase, thereby inhibiting the phosphotransfer of the regulation pathway. In general, it is unlikely that a GGDEF domain will have a similar function to a periplasmic sensor domain, because the former is involved in response regulation rather than perception. Therefore, which region of CqsS protein perceives autoinducers? What is the function of the response domain if the sensor histidine kinase domain performs similar functions to LuxQ? These problems need further study.

Homologous modeling, assisted by other analysis tools, such as molecular docking, is used to explain the mechanisms of toxicity of chemicals [46,100,101]. For Q67, which is increasingly used in monitoring environmental pollution and assessing toxicity, the luminescent inhibition rate for luminescent bacteria is always used as the endpoint. It is imperative to study the process of luminescence regulated by QS in Q67. However, it is difficult to analyze the crystal structures of proteins one by one. Even for the widely studied *A. fischeri* and *V. cholerae*, not all the crystal structures have been analyzed. Therefore, analysis from the perspective of homology modeling is the most appropriate. Although testing chemicals may simply reduce or improve the viability of bacteria in a way unrelated to QS, there is no doubt that some special chemicals, such as QS inhibitors, will directly affect the signaling pathway. For example, Sun et al. described the effects of QS inhibitors on nonpathogenic marine bacteria such as *A. fischeri* [102]. In addition, the action of other chemicals on the QS signaling pathway cannot be ruled out. Therefore, the construction of the predicted three-dimensional structures of proteins in the QS signaling pathway is an important step in toxicology.

It is worth noting that this study also indirectly reviewed the crystal structure analysis and functional analysis of proteins related to bioluminescence regulation by QS systems in other strains or species. In homology modeling, it is necessary to perform comparisons with a large number of existing crystal structures in the database. The protein with the highest or one of the highest sequence identities was selected as the template for constructing the homologous model. It can be seen from Table 1 and Table 2 that reliable templates were often from the same types of proteins of other strains. In addition, through structural and functional analysis, we found that nine proteins, including LuxI, LuxR, CqsA, LuxS, LuxPQ, LuxO, Hfq, HapR (LitR), and bacterial luciferase (LuxA and luxB), were widely studied among these QS-related proteins. However, the crystal structures of some of these proteins were incomplete, such as for LuxQ and LuxO, which is also why the template does not completely match the target protein (aligned positions in Table 1 and Table 2). Compared to these highly studied proteins, other proteins have drifted out of researchers’ fields of vision. Both LuxU and LuxD have only one crystal structure, and the crystal structures of the remaining six proteins (LuxM, LuxN, CqsS, LuxE, LuxC, and LuxG) have not been reported. Although we analyzed the transmission of signal and the regulation of bacterial luminescence in the QS system as much as possible according to the crystal structures and function from the existing reports, many questions around the putative functions of bioluminescence for bacteria remain unanswered. Research on these proteins, including crystal structure, will break through our current knowledge frontier.

## 4. Conclusions

In the study, the manner in which the QS pathway of Q67 regulates bioluminescence at the molecular level was analyzed by homologous modeling built by MODELLER. Firstly, through the analysis of four groups of autoinducer synthetase sensors (LuxI-LuxR, LuxM-LuxN, CqsA-CqsS, and LuxS-LuxPQ), the synthesis and binding of AIs, the initiation of the QS pathway, was explored. Then, we analyzed the involvement information of individual amino acids and specific structural elements in phosphotransferases (LuxU and LuxO), RNA chaperone (Hfq), and Master QS regulators (HapR) during signal transmission. Finally, we discussed how the individual amino acids and specific structural elements of enzymes, including luciferase (LuxAB), long-chain fatty acid reductase (LuxCDE), and flavin reductase (LuxG), were involved in the bioluminescence reaction. Unfortunately, many questions revolving around the putative functions of bioluminescence for bacteria are still unanswered because of the lack of availability of the crystal structure.

## Figures and Tables

**Table 1:** Modeling of protein monomers in QS pathway of Q67.

No.	Target Protein	Template Protein	PDB ID	Sequence Identity	Crystallo-Graphic Resolution	Aligned Positions	Normalized DOPE Score	GA341 Score	Predicted Structure Display
1	LuxI	LasI	1ro5-A	44%	2.3Å	1–193/193	−114.5	1	(a-1) in Figure 2
2	LuxR(VanR)	CviR	3qp6-A	32%	2.0Å	1–240/240	−102.2	1	(b-1) in Figure 2
3	LuxM	TofI	3p2h-A	**10%**	2.0Å	1–398/398	−79.5	0.5281	(c-1) in Figure 2
4	LuxN	WalK	4u7o-A	**26%**	2.4Å	450–683/859	−99.0	0.9981	(d-1) in Figure 2
5	CqsA	CqsA	3kki-B	68%	1.8Å	1–393/393	−120.1	1	(a-1) in Figure 3
6	CqsS	Histidine kinase	2c2a-A	**25%**	1.9Å	180–413/683	−97.8	1	(b-1) in Figure 3
7	CqsS *	Histidine kinase	2c2a/3luf	**25%**/**21%**	1.9Å/1.8Å	180–683/683	−101.8	1	(b-2) in Figure 3
8	LuxS	LuxS	5e68-B	72%	1.6Å	1–172/172	−110.1	1	(c-1) in Figure 3
9	LuxP	LuxP	1zhh-A	65%	1.9Å	39–386/386	−123.9	1	(d-1) in Figure 3
10	LuxQ	LuxQ	1zhh-B	37%	1.9Å	23–243/820	−101.5	1	(d-2) in Figure 3
11	LuxU	LuxU	1y6d-A	50%	--	1–112/112	−85.7	0.9915	(a-1) in Figure 4
12	LuxO	LuxO	5ep0-A	73%	1.6Å	1–393/468	−118.8	1	(b-1) in Figure 4
13	Hfq	Hfq	3rer-E	95%	1.7Å	2–65/88	−101.0	1	(c-1) in Figure 4
14	HapR(LitR)	SmcR	3kz9-A	81%	2.1Å	1–205/205	−133.3	1	(d-1) in Figure 4
15	LuxA	LuxA	3gfc-A	84%	2.3Å	1–355/355	−121.0	1	(a-1) in Figure 5
16	LuxB	LuxB	3gfc-B	61%	2.3Å	1–324/324	−122.9	1	(a-2) in Figure 5
17	LuxC	Retinal dehydrogenase 1A1	5abm-A	**16%**	1.7Å	1–413/481	−97.8	0.9542	(b-1) in Figure 5
18	LuxD	LuxD	1tht-B	69%	2.1Å	1–317/317	−123.7	1	(c-1) in Figure 5
19	LuxE	Acyl-CoA synthetase	4rvn-A	**19%**	2.2Å	1–384/384	−93.5	0.6242	(d-1) in Figure 5
20	LuxG	Fre	1qfj-A	36%	2.2Å	1–235/235	−124.1	1	(e-1) in Figure 5

*: Chimeric protein based on multi-template modeling --: Template proteins were obtained by the solution NMR method. Those with sequence identity less than 30% are shown in **bold.**

**Table 2:** Modeling of protein oligomers in the QS pathway of Q67.

NO.	Protein	Template	Sequence Identity	Crystallo-Graphic Resolution	Aligned Positions	Normalized DOPE Score	GA341 Score	Predicted Structure Display
1 **	LuxR(VanR)	3szt-AB	30%	2.0Å	1–240/240	−113.8	1	(b-2) in Figure 2
2	LuxN	4u7o-AB	**24%**	2.4Å	450–683/859	−106.2	0.9998	(d-2) in Figure 2
3	CqsA	3kki-AB	68%	1.8Å	1–393/393	−131.5	1	(a-2) in Figure 3
4	LuxS	5e68-AB	72%	1.6Å	1–172/172	−122.5	1	(c-2) in Figure 3
5 *	LuxPQ	1zhh-AB	65%/37%	1.9Å	43–386/386 23–243/820	−119.0	1	(d-3) in Figure 3
6	Hfq	3rer-ABCDEE	95%	1.7Å	1–65/88	−128.9	1	(c-2) in Figure 4
7	HapR(LitR)	3kz9-ABCD	81%	2.1Å	1–205/205	−141.4	1	(d-3) in Figure 4
8 *	LuxAB	3gfc-AB	84%/61%	2.3Å	1–355/355 1–324/324	−129.5	1	(a-3) in Figure 5
9	LuxC	5abm-ABCD	**16%**	1.7Å	1–413/481	−99.7	0.9335	(d-2) in Figure 5
10	LuxD	1tht-AB	69%	2.1Å	1–317/317	−121.9	1	(b-2) in Figure 5
11	LuxE	4rvn-AB	**19%**	2.2Å	1–384/384	−93.9	0.7494	(c-2) in Figure 5
12	LuxG	1qfj-ABCD	36%	2.2Å	1–235/235	−124.3	1	(e-2) in Figure 5

*: heterodimer three-dimensional protein. **: oligomeric template different from the monomeric template. Those with sequence identity less than 30% are shown in **bold**.

## Data Availability

Data supporting reported results can be found in Supplementary Materials or can be obtained from Shu-Shen Liu upon request.

## Supplementary Materials

The following are available online at https://www.mdpi.com/article/10.3390/biology10070638/s1, Code S1. The program code of model building and loop refining of LuxI based on 1ro5_A by MODELLER. Table S1. FASTA sequence of 19 coding sequences. Table S2. Model assessment of protein monomers in QS pathway of Q67. Table S3. Model assessment of protein oligomers in QS pathway of Q67. Figure S1. The residue alignment between target protein and the template by MODELLER where the symbol “*” indicates that the corresponding residues are highly conserved. Figure S2. Ribbon diagram of protein and template monomers in the QS pathway of Q67 where loop, α-helices, and β-sheet are shown in silvery-white, red and green, respectively. “N” and “C” refer to the N-terminal and C-terminal of the protein monomer. Figure S3. Ribbon diagram of protein and template oligomers in QS pathway of Q67 where colored ribbon diagram refers to chain A, yellow to chain B, blue to chain C, green to chain D, black to chain E, red to chain F. “N” and “C” refer to N-terminal and C-terminal of the protein oligomer. Figure S4. Sequence and topology diagram of 17 functional protein models where different chains are separated by symbols ‘┍’. Blue arrows refer to numbered *β*-sheets from N-terminal to C-terminal and orange column to numbered *α*-helices from N-terminal to C-terminal. Since subunit chains do not have exactly the same secondary structures in the template proteins, the ABCDEF after the protein name represents the A to F chains of the protein respectively. Specially, for CqsS monomer with double template modeling, region based on template 2c2a is colored by green and region is based on template 3luf is colored by blue. Figure S5. Ramachandran plot of 17 function proteins where green circles refer to residues in core regions (in blue closed line), green triangles to residues in additional allowed regions (in red closed line and outside blue closed line), red circles to residues in generously allowed regions (outside red closed line) and red triangles refer to residues in disallowed regions (outside red closed line).
