# Supplementary material for: Protein Model and Function Analysis in Quorum-Sensing Pathway of Vibrio qinghaiensis sp.-Q67
Source: Biology (Basel). 2021 Jul 9;10(7):638. doi: 10.3390/biology10070638 (PMC8301110; doi:10.3390/biology10070638)
Supplement: Supplementary file 1 [file biology-10-00638-s001.zip › biology-1278400-supplementary.pdf]

Code S1. The program code of model building and loop refining of LuxI based on 1ro5_A by MODELLER.

```
from modeller import *
from modeller.automodel import *        # Load the automodel class
log.verbose()
# Override the 'special_restraints' and 'user_after_single_model' methods:
class MyModel(loopmodel):
#def special_restraints(self, aln):
# Constrain the A and B chains to be identical (but only restrain
# the C-alpha atoms, to reduce the number of interatomic distances
# that need to be calculated):
#s1 = selection(self.chains['A']).only_atom_types('CA')
#s2 = selection(self.chains['B']).only_atom_types('CA')
#self.restraints.symmetry.append(symmetry(s1, s2, 1.0))
def user_after_single_model(self):
# Report on symmetry violations greater than 1A after building
# each model:
self.restraints.symmetry.report(1.0)
env = environ()
# directories for input atom files
env.io.atom_files_directory = ['.', '../atom_files']
# Read in HETATM records from template PDBs
env.io.hetatm = True
# Be sure to use 'MyModel' rather than 'automodel' here!
a = MyModel(env,
alnfile='../step3_align/LuxI-1ro5A.ali',
knowns= '1ro5A',
sequence='LuxI',
assess_methods=(assess.DOPE,
# soap_protein_od.Scorer(),
assess.GA341))                    # code of the target
a.starting_model= 1                    # index of the first model
a.ending_model    = 20                 # index of the last model
# (determines how many models to calculate)
# Very thorough VTFM optimization:
a.library_schedule = autosched.slow
a.max_var_iterations = 300
# Thorough MD optimization:
a.md_level = refine.slow
# Repeat the whole cycle 2 times and do not stop unless obj.func. > 1E6
a.repeat_optimization = 2
a.max_molpdf = 1e6
a.loop.starting_model = 1              # First loop model
a.loop.ending_model      = 2           # Last loop model
a.loop.md_level            = refine.fast     # Loop model refinement level
a.make()                               # do comparative modeling
```

Table S1. FASTA sequence of 19 coding sequences.

No.	protein	FASTA sequence
1	LuxI	MTISIYSHTFQSVPQADYISLLKLRYKVFSQRLQWELEINQGMETDEYDVPEAHYLYAK-<br>EAQGHLIGCWRILPTTSRYMLKDTFPELLGVQQAPKAKEVYELSRFAV-<br>DKDHSAQLGGVSNVTLQMFQSLYHHAQQNHIHAYVTVTSASVEKLIKRMGIP-<br>CERLGDKKVHLLGSTRSVALHIPMDEAYRTSVNA
2	LuxR	MQKILRLIQENQQITSHNDLENVLNGLNNLIDHEFFLFGLSFQPTLKTSETLITDNY-<br>PPSWRQQYEQSGFVHVDPIVKYSMANFLPIRWDDAKRVNEDGRIIFEEARING-<br>LKTGFSIPIHGLRGEFGMISFATSDTKSYDLNQQSIHTSQLIVPLLAHNIDNI-<br>AHSHKNVSPRVVLTAREVQCLTWAAEGKSAWEIATIINTSERTVKFHFSNTCKKL-<br>GATNRYQAITKAILGGYINPYL
3	LuxM	MKIIPSLGSQLATSLPIEKKQRALIEFVINTYQPQQRADLFRAVTDCRKNQLLNLF-<br>PEHHNKSFSILFELMDYRDLIQRYPNTLDEEIAHLEQAVSECYSHWLDFWCECEIAAIK-<br>TLFPIEADAPPRIELPLKDCAYRGFLIDQIEHSELWVTTPSHPQKMPIKDAITLSNLELF-<br>IKGEKWYEMLPLLSLSQKGKHFVLLKHPDNEASPT-<br>LVASMLVKDWSVNQTWLSYAPQFSNEQWLFCLPDHGYHALMELQLFK-<br>PALSKCDSLPKFDQQFRRQLTDTRAVCEVLRFTVSGNAQQKLYFLY-<br>LAQKKFMHVMHQLGYKLGLFATAQSFMLNFYQAVDPKAYFHCGNCDLNSDGVKTYR-<br>GFWNFESMDRVFRDADFRDYKHAVSQSRKYHLIKREEYA
4	LuxN	MLDFSLEPILYPKAITLIVAVAMVLVWLTYYCYRLKQKNEMIWGTRHVAYIAYSLFI-<br>IAWIGSNAYFHTDWLAELGADRAIFMAKFANVVSSFAFAFAYYFSCQLSAEQRGGRI-<br>YLWQQLFFIAIVMYFLVINLQPNLTVRHVDIAGPSKFVIEFGPHTPYFFSALL-<br>CAMIMTLFNLISMRANSSKLTLAKTNYMIAGILVYMLSTLILQVGITFFFQDFSLTWLPPAL-<br>SISEMMFVGYALITSRFYSVKYLAYLCLNTALVCGVLFIPLGAIFIPLTDSNQWLVAIPL-<br>CALIGITWNPLYKRLSRYASLLIYGNQQTPVEQILALEDDFKRSIDDAMRRLSQLLY-<br>IADDKLQFVNSNYNETVYERYLSSKQTALVFDELFEKLDNKTAAKNSIKALY-<br>DKMSSNNTALVMPLFGHSKLVTHLLISPHKINNQMFSNEEIAALQTLLTRIQSI-<br>IEADRRVCQSRALANSIAHEMRNPLAQVQLHFEILKQHIDNQAPAQQIKQDIENGQAAI-<br>QRGRQLIDIILREVSDSSPEHEPITMTSIHKAVDQAVSQYGFENEKVIERIHLPQQDD-<br>FVAKLNETLFNFVIFNLIRNAIYYFDSYPNSQIEITTQIGTYENILIFRDTGPGID-<br>DVISYKIFDDFFSYQKSGGSGLGLGYCQRVMRSFGGRVECK-<br>SKLGEFTEFHLYFPMVPNAPQADSLRTPDFKGWQQPKTNTEQRTVDDIQPIDQPFLINN-<br>KAPTVLIVDDKEVQRSLVQMYLNQLGVNNLQANNGENAVEIFKANCID-<br>LILMDIQMPVMNGFEASQIIKAHSPQIPIIALSGESGERELEMISKLMDGRLEK-<br>PTSLNALRQVISHWLNKDVVLNAHTAKSGTVT
5	CqsA	MNSRKWKHELPTSIQSRIDYFINDLINANANGKHLVLGKRPTKNDI-<br>VLQSNDYLSLADHPLIKARLKNAIDKTHDSVFMSAIFLQDDESKPS-<br>LETKFAEFLGFDSCLLSQSGWNANIALLQAVCGPQSNVYIDFFAHMSMWEGARYA-<br>NANIRPFMHNNCEHLQKLIARHGPGIIVVDSIYSTIGTVAPLT-<br>DLVTIAKEWGCAILVDESHSLGTHGKKGAGLVNELGLTSEVDFITASLAKTFAYRAGAI-<br>WANNNVNHCIPFVGYPAIFSSTILPYEVEALEATLDIIKSADAKRSQLHRNAHTLRRGLN-<br>SIGLSVRSESQIIALETGDERNTEKVRDYLEDNQIFGAVFCRPATAKNKNIIRLSLNSDVTD-<br>SQIDTILTVCDKANKESSLYFK
6	CqsS	MDVVKKIYQYAEPNLTLVGWMGFIGFPLYYYVWTYLFPQPYESIELRVICSVFFAGII-<br>FRNSTPKHLQRYLPYYYLFSIGFCLPFFFGYMMLMNDWSTIWAMSFMASI-<br>FLHILLVYETKVMLIQAAVALACAYFVTYGVMNAELRVVVEWPYVPIF-<br>LFTYIFGNQFYFRNQIEHEAKVSIAKSFGAGIAHEMRN-<br>PLSALKASIEVMGSVLPSSTASKSESYTLPSKDLELITGLLRDADEVIRSGNET-<br>IDLLLTSIDQNRVSTSTFKKHTVKPVIEEALHSFSYKRPEDRQAVTLDVVDD-<br>FNFLGSSTLLKYVLYNLLKNAFYYKNNTMFHIHICMES-<br>KGNKPQITVKDNGVGIEPHLLDDIFKDFYTFGKNGSYGLGLPFCRKVMNAFGG-<br>DITCRSELGEWTEFTLRFPEYDSQAVANIKLDLMKAKSLLYIGKTSIVNRYLSEKSFYL-<br>GFQLNVLEPESVLLREEFEFEFDLIFVDLQTLHETPDYLT-<br>KLEPKLHFTQAKICYLYDQNQRYALTMERHLTIYPIEKHKLLLDGSAIIDELLFEAEELIAD-<br>KNVIPLKQYDYEKRILLVDDNHSLRS-

7	LuxS	MPLLDSFTVDHTRMHAPAVRVAKNMQTPKGDTITVFDLRFTAPNKDILSEKGIHTLEH-<br>LYAGFMRAHLNGHDVEIIDISPMGCRTGFYMSLIGAPTEAQVAQAWLAAM-<br>HDVLKVESQDQIPELNEYQCGTASMHSLTEAKEIAQAII-<br>TAGIAVNKNDELALPESMLKELKVD
8	LuxP	MSAKSAIVYHLIQKLVYENAMRKILFLSFVFSVSLAASTQAAAQVLNGY-<br>WRYQEFLDLNPAQQVLTDQMIQAVRQPPAALSIEQDKPIHISVVYPGQQVSDY-<br>WARNIKAFEMRLDELGIRYQINQVFTRPNIDIRQQSLSLMEAIRNKSDYLVFTLDTIRHRK-<br>FIEQLLSSNQTKIILQNITTPVKSWGDRQPFMYVGFDHEIGTMKLVDYYKKSVP-<br>KNSAYSVLYYSEGYVSDARGDSFIQEMSRDSKYHLASSFYTKATRD-<br>SAFDATLHILKQTPDINFIYACSTDVALGAMDALQQQNREDITLNGWGGGE-<br>AELEALQNSTLDVTIMRVNDDAGVAMAEAIKWDLEGKPVPTVYSGDFELVTKEASSAY-
9	LuxQ	MTLSVLFQSYQISSKIIAQEVQRTASQTSSLIQNLFNFRLSLLQVNQDSSAKNTGLVKAIR-<br>KGDNDSIDQFFFGVDQLEVTNTPDIRFISDSDSLMWEDGNAQFY-<br>GIEQNELVNMSHKVALSNNWHMIKSPSQLGYTYLMLRRTPVIDSPSGEV-<br>IGFLHLASVLNNNYVLAETLREGSNSENLVMTTGDKVLASTINGDDTY-<br>TEHDLLDDNFDAYSFADYMLSRTELTTEGVPIYINVLSVQSNKNVLQLRENYFFWMI-<br>FALFAMVIVSILTRWWLHRRVKIEISSLMNYTRKASEAGNYELFSGSKIYEFDHFGRTLEHT-<br>FQRLSEQEKQFEDLFNFSLSPIIVWSIDGDIIKMNPSAKKYFMRRNDQQQCIFDALKQEL-<br>QPKIRLAVEQETFREINVEVGNKVFRWNLSPIIFEQRIESIITQGQDITTIAEAAKQSR-<br>LARQEAEESARIRAEFLAKMSHELRTPLNGILGVSQLLKREISNHEQLEQVNVLCSSGEH-<br>LLAVLNDILDFSKIEEEQFRIQESDFKLVEVISAVESIYRPLCAEKEIEFSLK-<br>SNIPPDMMIHGDQVRLNQILFNLLNNATKFTHNGLISIEFQQKNSSHLLISISDT-<br>GIGIREQDLALIFEPFTQVESNTTREYGGSGLGLAIVKSLIEMLDGTVSVKSQFGVGTT-<br>FSIKLPLVAVSGCKKEVTSYASTPPHELFEKPIKVLLVEDNHTNAFIAQAFCKKYR-<br>MEVSRVEDGLQAIDYLKDHPVDLILMDNQLPYLGGVETTH-<br>LIKHELMMDVPIYACTADGMQSTNEAFMAAGAEYVIVKPIKEKALHEAFCHFQKRQK
10	LuxU	MELINKNKIAHLAQEIGEENVPILLDIFLSELSAYTQKLADQNLPD-<br>KIAYLKDISHALKSSAASFGADRLCAKAVDIDSKGKANCIFDEAEEVAAMRALI-<br>EETHRCYCHLMD
11	LuxO	MQPDFSLQKTKYLLMVEDTASVAALYRSYLTPLEIDINIVGT-<br>GRDAIESLAHREPDLILLDLRLPDMTGMDVLHAVRNQYPNVPVIFMTAHG-<br>SIDTAVEAMRHGAQDFLIKPCEADRLRVTVNNAIRKASKLKNEADNPGNQNYQGFIGSS-<br>QTMQSVYRTIDSAASSKASIFITGESGTGKEVCAEAIHAASKRGDKPFIAIN-<br>CAAIPKDLIESELFGHVKGAFTGAAT-<br>DRQGAAELADGGTLFLDELCEMDLDLQTKLLRFIQTGTFQKVGSSKMKSVDVRFVCAT-<br>NRDPWKEVQEGRFREDLYYRLYVIPLHLPPLRERGDDVIEIAYSLLGFMSKEEGKGFVR-<br>LAPEVVERFTRYEWPGNVRQLQNVLRNVVVLNHGNEIELSMLPPPLNQVVDNKIRLAN-<br>LTTETRKENITVHDIFPLWMTEKQAIEQAIEACDGNIP-<br>KAASYLDVSPSTIYRKLQAWNSRENS
12	Hfq	MAKGQSLQDPFLNALRRERIPVSIYLVNGIKLQGQIESFDQFVILLK-<br>NTVNQMVYKHAISTVVPARAVSHHTAEPRTGSDRPSDKSEE
13	HapR	METSIEKRHRTRLSPQKRKLQLMEIALEVFATRGIGRGGHADIAEIAQVSVAT-<br>VFNYFPTREDLVDDVLTHVVRQFSNFLADNIDLDLHAKDNLTNLTTEMIS-<br>LVIEDCHWLKVWFEWSASTREEVWPLFVSTNRTNQLLVQNM-<br>FIKAIERGEVCDQHDPEHLATLFLGIFYSLFVQANRVRDEDSMNALVKSYLSMLCIYKKDH
14	LuxA	MKFGNFLLTYQPPGLSQTEVIQRLVNL-<br>GRASESCGFEAVWLLEHHFTEFGLLGNPYVAAANLLGATKHLHVGTAAI-<br>VLPTAHPIRQLEDVNLLDQLSKGRFRFGICR-<br>GLYDKDFRVFGTDMNNSRALMDCWYDLITTGMTQGTVSADNEHIHFPEVKVQPAPY-<br>HRSGAPVYVVAESASTTEWAAKRGLPMILSWIINTHEKKAQLDLYNEIALEHGH-<br>DIQNIDHCLSYITSVDHDSQHAKDICRQFLAHWYDSYVNATRIFDDSDQTKGYD-<br>FNKGQWRDFVLKGHRDTNRRIDYSYEINPVGTPEECIRIIQQDIDTTGITNIC-<br>CGFEANGSEQEIIASMKLFQAEVMPRLKNPN
15	LuxB	MKFGLFFLNFLHSGQSCTEVFDAMIDSVNYAEKGHFDRLFI-<br>YENHFNDHGIVGAPLTAASFLLGMTERIKVGSLNHVLTTHHPVRTAEETGLLDQMSQGR<br>FILGFSDCENRDEMIFFNRPLDSQQPIFEACYQIINDALTSGYCHPDNDFYSFPKIS-<br>VNPHSYTQGGPQQYVYASSTQVVGWAAKRALPLTFKWDDSNTTRQQYARHYRETAKKY

		GVDVQAVRHQLALLIN-QNDDGEIARIEARQYLTQYVIERYPSDEIEIVLERIIKESAIGTYEESTQAARMAIEMCGASDLLISVESIKEPAHRLHVLDVINSNIAKYHQ
16	LuxC	MTKHIPFIINGKISTVSKLEKPELNSVIFREKTLDLDILTDHITDEIIDHKKTRELT-LNNIVNFLYTVGQRWKNEEYTRRRSYIRDLKNYLGYSEEMAKLETNWIAMILCSKSALY-DIVQTELGSRHILDEWIAQDECYVKALPKGRSLHLLAGNVPLSGVTSILRAILTKNQIIV-KMSSNDPFTPHALAMSFIDVDPNHPITQSISVIYWPHTQCTQVAQRLMQKMDVVVAW-GGSEAIRWAAEHTPSHAELIKFGPKKSLTIINDPENLIEAAEGAAHDICFYDQQACF-STQNLFYLGSRFPEFKQALREQLQRYARILPKSQSSIDEQADFSLTLRECQFAG-FTSEMGSQQDWMMIESPAGTELNHPLGRCIYLHQMASFEEILPFVIKGQTQTVSLF-PWSCSFQYRDQLAAHGAERIVESGMNNIFRVG-GAHDVMRPLQRLVRFISHERPSHFTTKDVAVDIEQTRYLEEDKFLVFVP
17	LuxD	MSHRSHWEVDEPCTIDHVISLSGNQQIHVWETPPILKFNQMPTRRNSILIASGFARRMDH-FAGLARYL-SANGFHVYRYDSLHHVGLSSGTIDQYTMSIGKQSLEIVMAWLNNRGVTDVGVI-AASLSARIAYATVNELDLSFLISAVGVVNLRDTLEKAFGFDYLSLPIQDLPNDLDFEGHKL-GAEVFVRDCFANNWDSFSSTVEQMRHLSIPFIAFTANGDDWVKQDEVI-ELLSQLPAGQSKLYSLLGSSHDLGENLVVLRNFYQSVTKAALALDSNSFDIDIPFVEPT-
18	LuxE	MDTTLPLIDKQAVDKFDIEASSYLDDLIFMSEPNSWSYEEQEKIRHDVIMKAFR-WHYQNNTDYRRYCQTIGIGLEIEHLDDIPVYPTSIFKTMRVTSAKPQEI-EHWFTSSGTQGQKSHIPRDRLSIERLLGSVNYGMKLVGAWFDHEMELVNLGPDRF-NAHNIWFKYVMSLVELLYPTAFTAKDDLVDFDQTLMHLYRIQSMGKTTCLIGPPYFVYLL-CQHMKAEKIHFRAGHKLYIITGGGWKTHENSALNRDQFNQLLMETFGLNDIS-QIRDTFNQVELNTCFFEDDQQRKCVPPWVYARALNPRTLQPLPDGEIGLMSYMDASAT-SYPAFLITDDLGYIHQHTEKIAYTTVQIVRRINTRAQKGCALKMSQHFSFPQITPQ
19	LuxG	MKFSCHVHTVQHLAPGIYQIILAPHYPLDFKAGQFLKLTLAGKDRYFSIASCPSQPG-FIELHIGTSKTDEGILSTIAALHEFKEAELPLEIEGPLGNAWLRKESNNPILFIAGGT-GISYIMSLLRNALHNQLDQSIYLYWGVKGINQLYLHPELLMLSDQYP-NLHYVCSLEESCDQIMSREGLVVDAILNDFSDLQDFDIYLCGPINMIKEGKKYLLEKC-NATMGNMYGDGLAYV

Table S2. Model assessment of protein monomers in QS pathway of Q67.

**No.**	**protein**	**Verify3D**	**ERRAT**	Prove	Ramachandran plot*			
					core	allow	gener	disall
**1**	**LuxI**	**96.37%**	**87.57**	**5.4%****	85.1%	12.6%	1.1%	1.2%
2	VanR	80.42%	**70.69**	4.0%	90.3%	9.3%	0.4%	0.0%
3	LuxM	**27.89%**	**6.92**	**15.5%**	70.7%	20.8%	5.5%	3.0%
4	LuxN	**40.60%**	88.50	**5.6%**	91.4%	5.2%	2.4%	1.0%
5	CqsA	84.48%	90.65	3.9%	91.9%	7.3%	0.8%	0.0%
6	CqsS	**53.42%**	**65.49**	**5.9%**	90.8%	8.7%	0.0%	0.5%
7	CqsS*	**46.43%**	**52.82**	**8.9%**	89.3%	9.2%	1.1%	0.4%
8	LuxS	**59.30%**	99.39	2.1%	92.8%	5.9%	0.7%	0.7%
9	LuxP	94.25%	86.14	3.7%	96.5%	3.5%	0.0%	0.0%
10	LuxQ	85.07%	**67.14**	**6.8%**	93.1%	5.9%	1.0%	0.0%
11	LuxU	**27.68%**	84.62	**7.3%**	89.5%	7.6%	2.9%	0.0%
12	LuxO	94.91%	87.24	4.8%	95.7%	4.1%	0.2%	0.0%
13	Hfq	**64.62**%	91.23	2.1%	94.7%	3.5%	1.8%	0.0%
14	HapR	82.93%	95.36	2.3%	94.8%	5.2%	0.0%	0.0%
15	LuxA	89.30%	91.18	3.3%	94.3%	5.4%	0.0%	0.3%
16	LuxB	93.21%	86.39	4.9%	94.2%	5.5%	0.3%	0.0%
17	LuxC	**50.61%**	**42.14**	**8.7%**	86.2%	9.8%	2.9%	1.1%
18	LuxD	85.49%	85.11	**6.2%**	88.9%	9.4%	1.4%	0.3%
19	LuxE	**59.11%**	**45.21**	**9.2%**	85.2%	11.6%	1.7%	1.4%
20	LuxG	96.60%	**61.06**	**6.4%**	90.1%	8.9%	1.0%	0.0%

*: core, allow, gener and disall refer to residue ratio in core, additional allowed, generously allowed and disallowed regions, respectively.**: The value that can't pass the evaluation parameter were shown in **Bold** (empirical limits of pass: greater than 80.00% for Verify3D, greater than 80.00 for ERRAT, less than 5.0% for Prove and greater than 90.0% for the proportion of residues in the core and additional allowed).

Table S3. Model assessment of protein oligomers in QS pathway of Q67.

num	protein	Verify3D	ERRAT	Prove	Ramachandran plot* core	allow	gener	disall
**1**	**VanR**	**74.58%**	**A: 71.98**<br>**B: 80.17**	**5.8%**	90.0%	8.3%	1.2%	0.5%
2	LuxN	**43.80%**	A: 81.25<br>**B: 71.36**	**5.7%**	91.0%	6.9%	1.4%	0.7%
3	CqsA	**79.90%**	A: 91.15<br>B: 92.19	3.8%	92.6%	7.3%	0.1%	0.0%
4	LuxS	**70.35%****	A: 95.12<br>B: 98.78	2.8%	94.1%	5.2%	0.7%	0.7%
5	LuxPQ	89.20%	A: 85.42<br>**B: 69.86**	3.9%	94.4%	5.2%	0.4%	0.0%
6	Hfq	**67.45%**	A: 100.00<br>B: 98.18<br>C: 91.07<br>D: 100.00<br>E: 96.43<br>F: 87.50	2.6%	93.2%	5.1%	1.8%	0.0%
7	HapR	**79.39%**	A: 93.91<br>B: 89.80<br>C: 84.70<br>D: 94.36	3.0%	97.4%	2.6%	0.0%	0.0%
8	LuxAB	89.25%	A: 94.22<br>B: 80.38	4.0%	93.9%	5.8%	0.0%	0.3%
9	LuxC	**46.00%**	**A: 40.74**<br>**B: 45.66**<br>**C: 52.35**<br>**D: 47.28**	**8.9%**	85.7%	11.6%	1.9%	0.9%
10	LuxD	81.86%	**A: 74.11**<br>**B: 74.43**	**7.1%**	88.7%	8.9%	1.9%	0.5%
11	LuxE	**48.05%**	**A: 40.16**<br>**B: 39.36**	**8.3%**	83.8%	12.2%	1.6%	2.5%
12	LuxG	94.04%	**A: 48.90**<br>**B: 54.63**<br>**C: 58.59**<br>**D: 52.86**	**6.1%**	90.8%	7.8%	0.7%	0.7%

*: core, allow, gener and disall refer to residue ratio in core, additional allowed, generously allowed and disallowed regions, respectively. **: The value that can't pass the evaluation parameter were shown in **Bold** (empirical limits of pass: greater than 80.00% for Verify3D, greater than 80.00 for ERRAT, less than 5.0% for Prove and greater than 90.0% for the proportion of residues in the core and additional allowed).

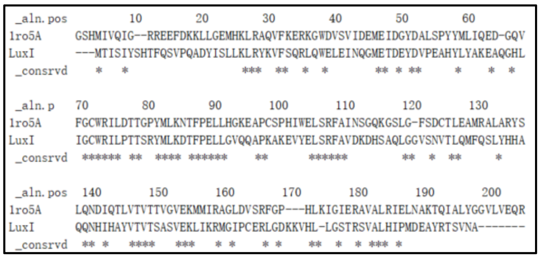

(1) LuxI *vs* 1ro5-A

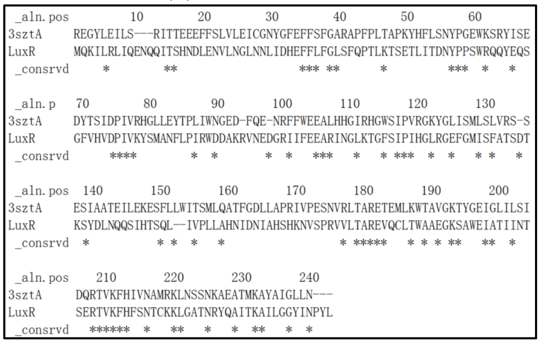

(3) LuxR(VanR) *vs* 3szt-A

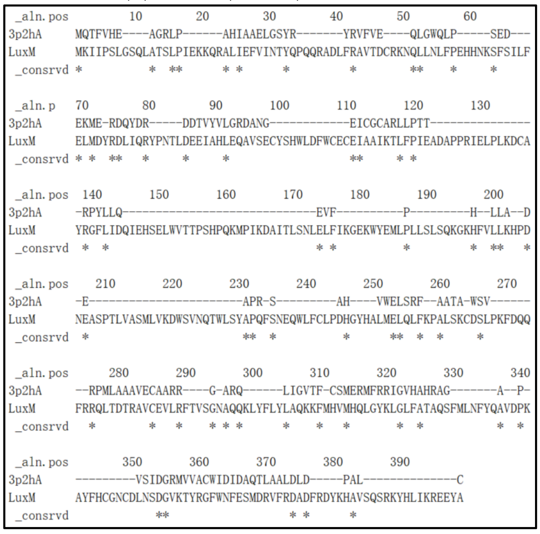

(5) LuxM *vs* 3p2h-A

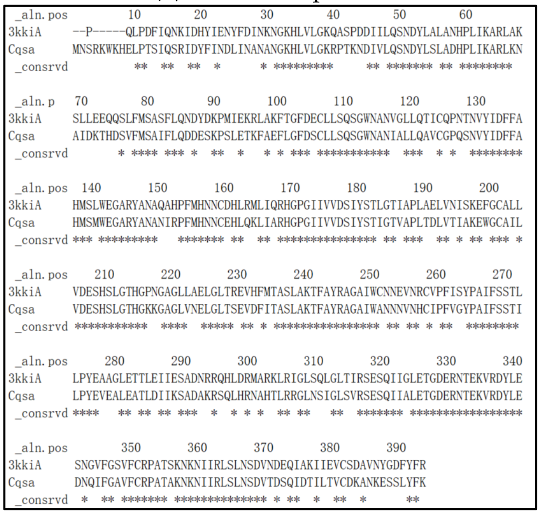

(8) CqsA *vs* 3kki-A

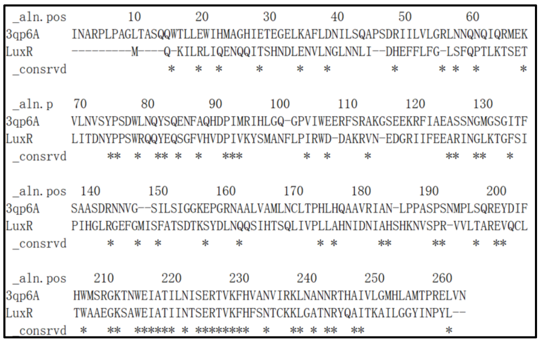

(2) LuxR(VanR) *vs* 3qp6-A

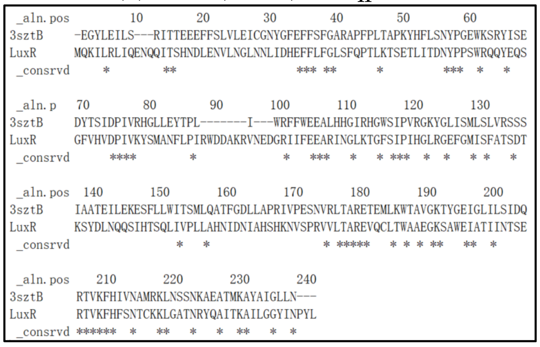

(4) LuxR(VanR) *vs* 3szt-B

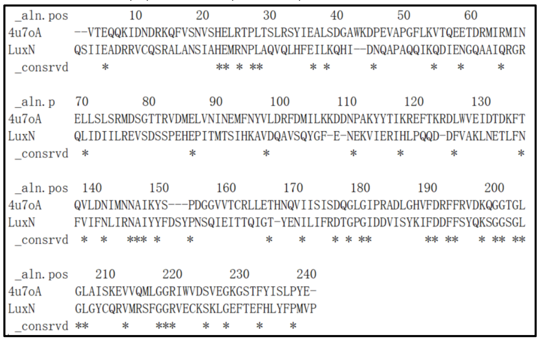

(6) LuxN *vs* 4u7o-A

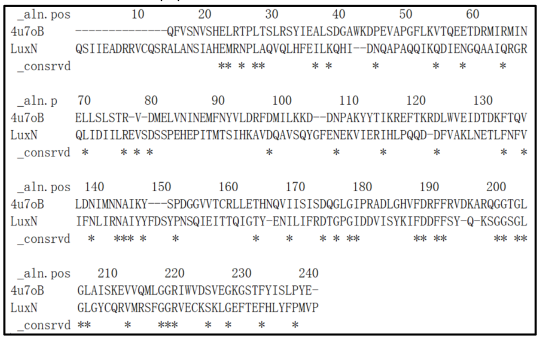

(7) LuxN *vs* 4u7o-B

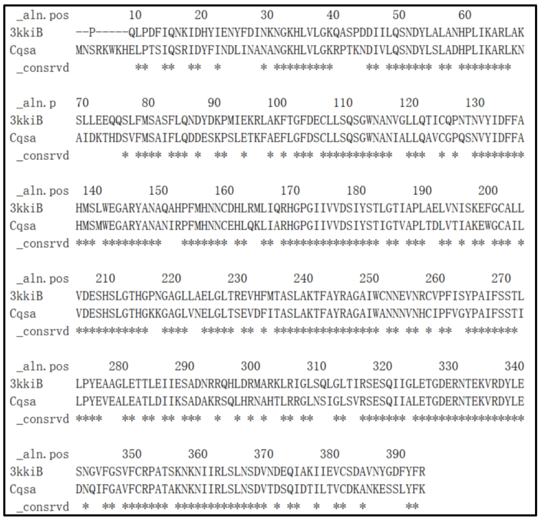

(9) CqsA *vs* 3kki-B

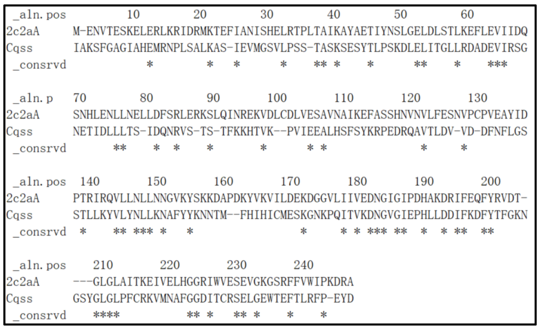

(10) CqsS *vs* 2c2a-A

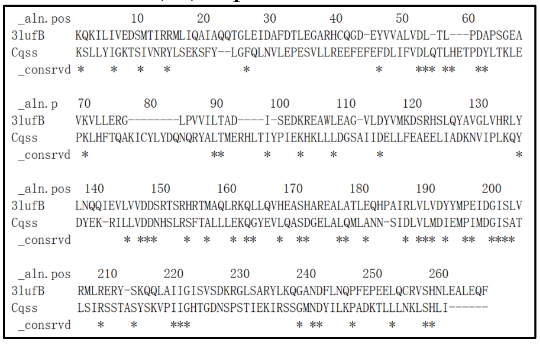

(11) CqsS *vs* 3luf-A

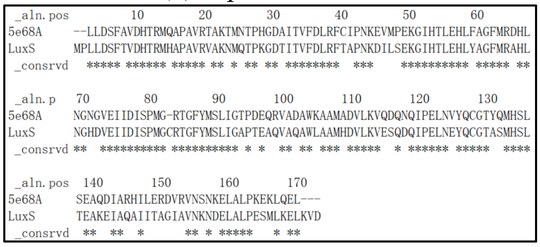

(12) LuxS *vs* 5e68-A

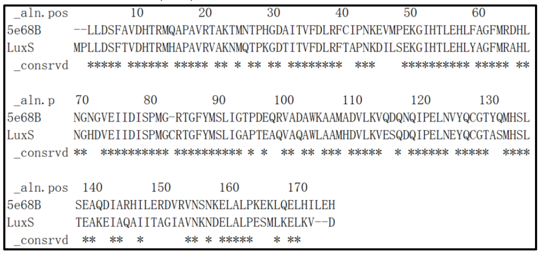

(13) LuxS *vs* 5e68-B

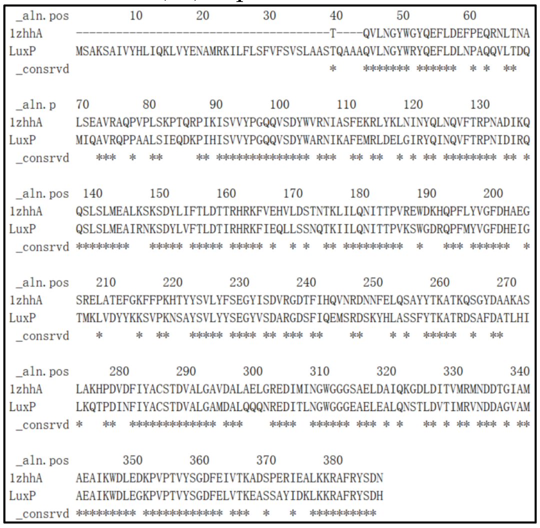

(14) LuxP *vs* 1zhh-A

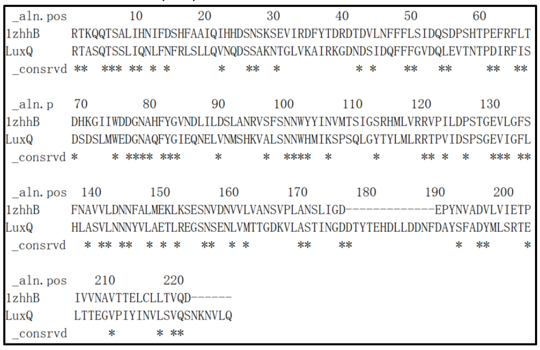

(15) LuxQ *vs* 1zhh-B

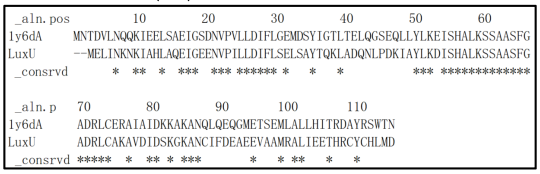

(16) LuxU *vs* 1y6d-A

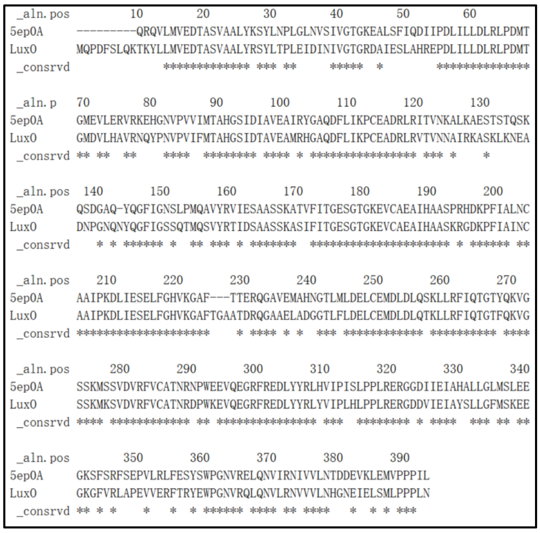

(17) LuxO *vs* 5ep0-A

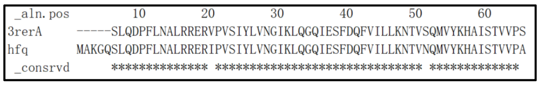

(18) Hfq *vs* 3rer-A

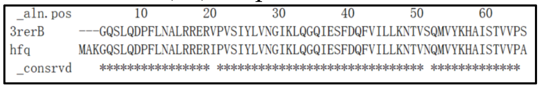

(19) Hfq *vs* 3rer-B

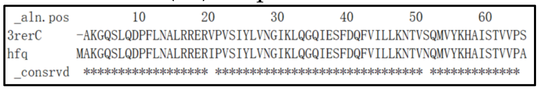

(20) Hfq *vs* 3rer-C

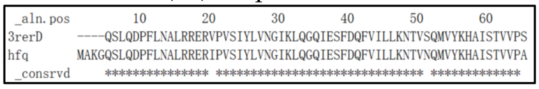

(21) Hfq *vs* 3rer-D

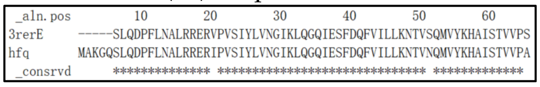

(22) Hfq *vs* 3rer-E

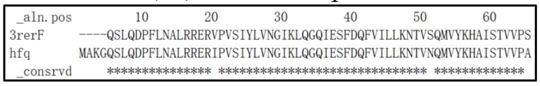

(23) Hfq *vs* 3rer-F

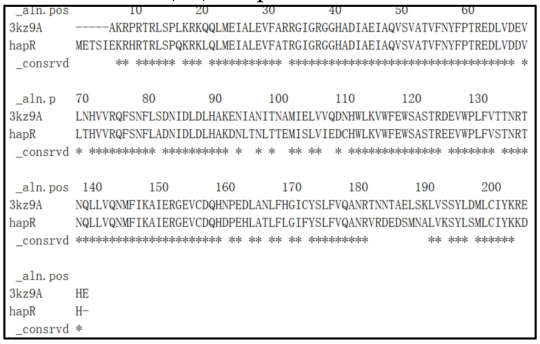

(24) HapR(LitR) *vs* 3kz9-A

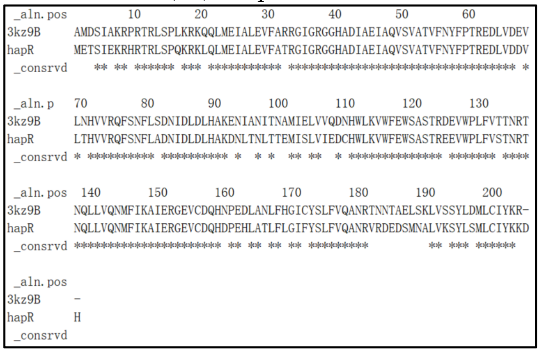

(25) HapR(LitR) *vs* 3kz9-B

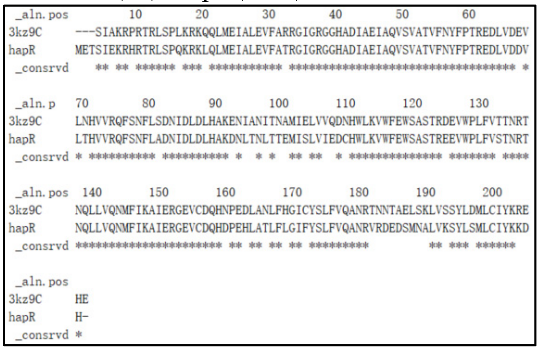

(26) HapR(LitR) *vs* 3kz9-C

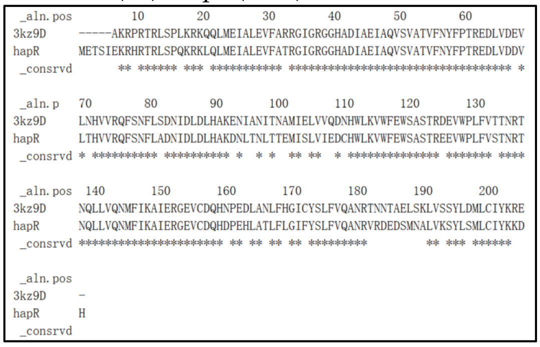

(27) HapR(LitR) *vs* 3kz9-D

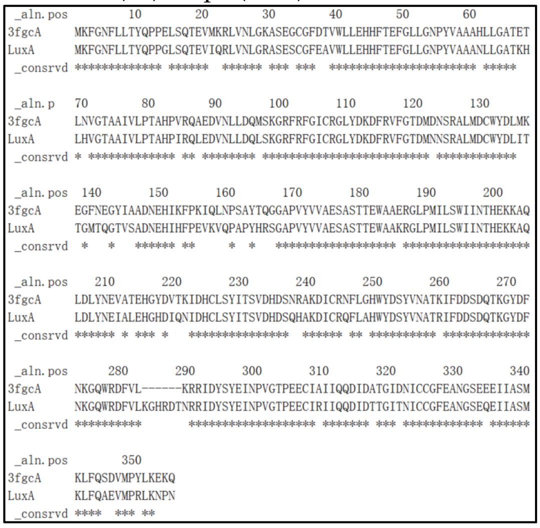

(28) LuxA *vs* 3fgc-A

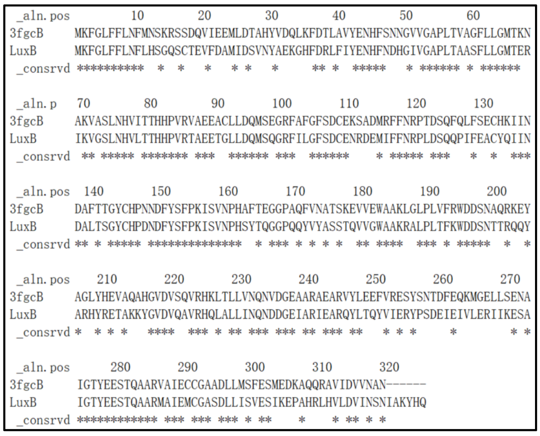
(28) LuxB *vs* 3fgc-B

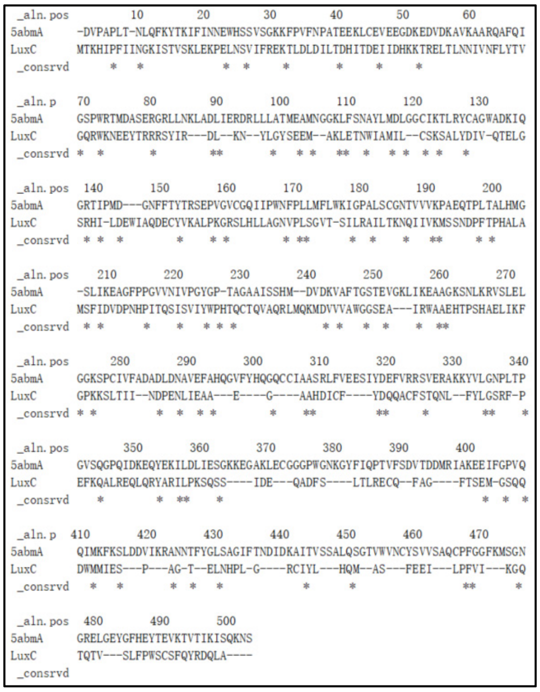
(29) LuxC *vs* 5abm-A

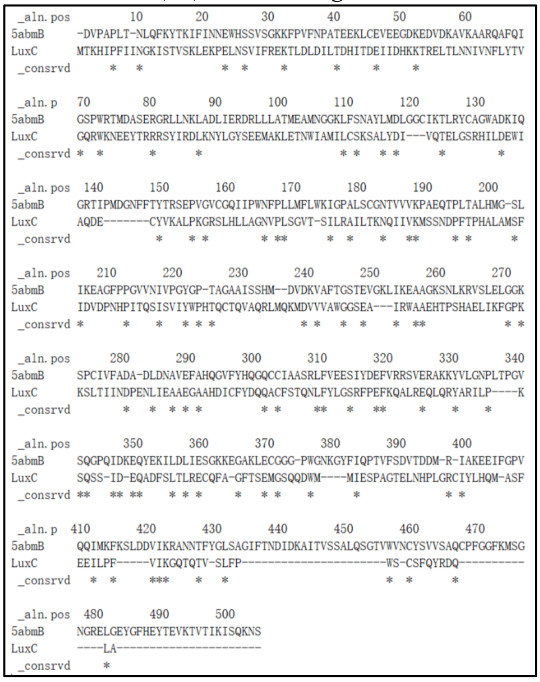
(30) LuxC *vs* 5abm-B

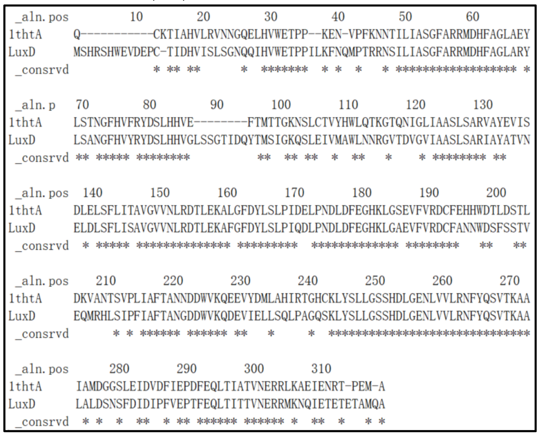
(31) LuxD *vs* 1tht-A

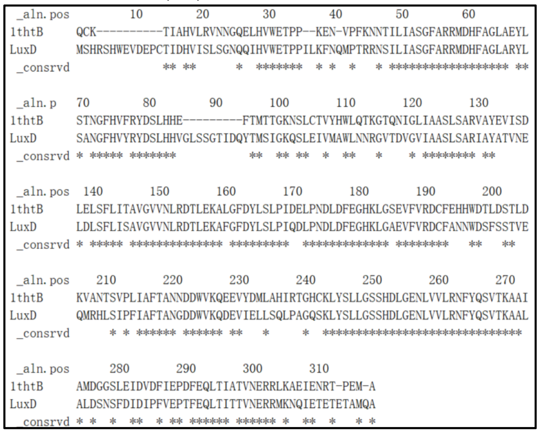
(32) LuxD *vs* 1tht-B

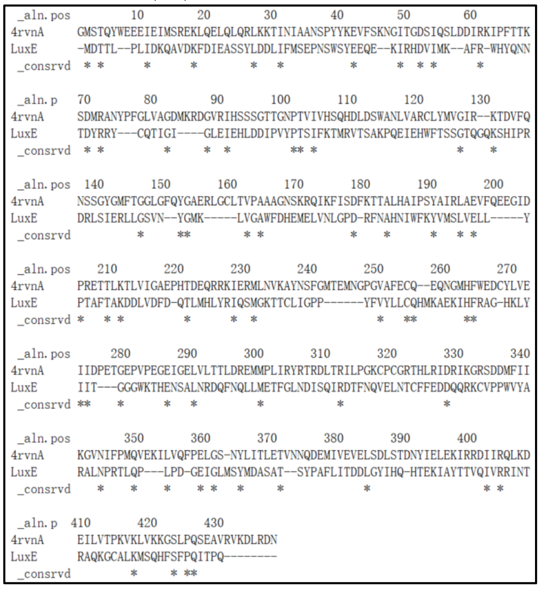
(33) LuxE *vs* 4rvn-A

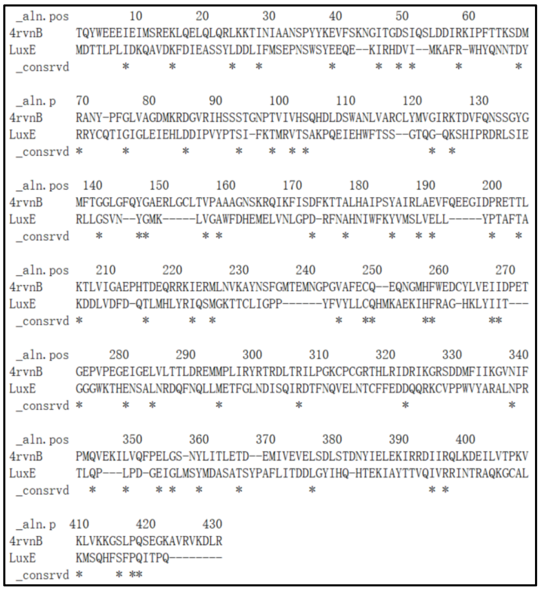

(34) LuxE *vs* 4rvn-B

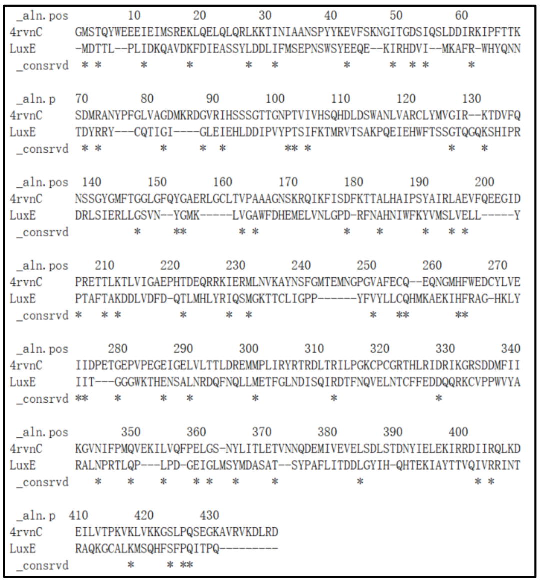

(35) LuxE *vs* 4rvn-C

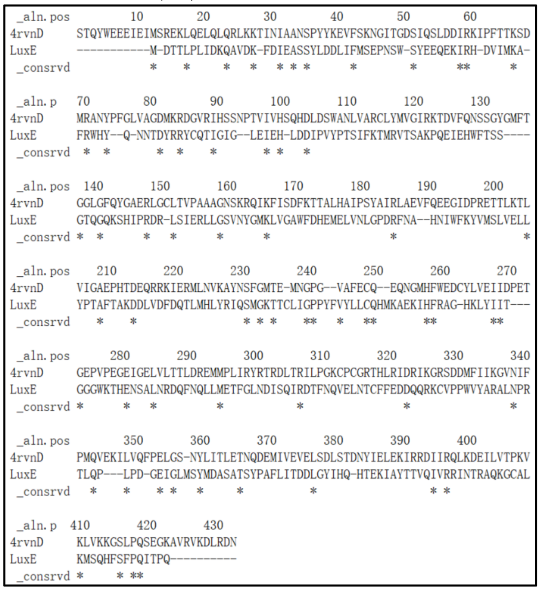

(36) LuxE *vs* 4rvn-D

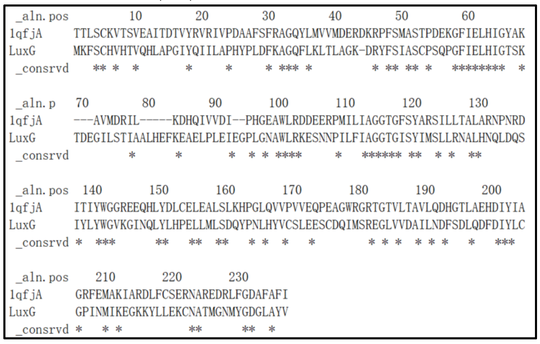

(37) LuxG *vs* 1qfj-A

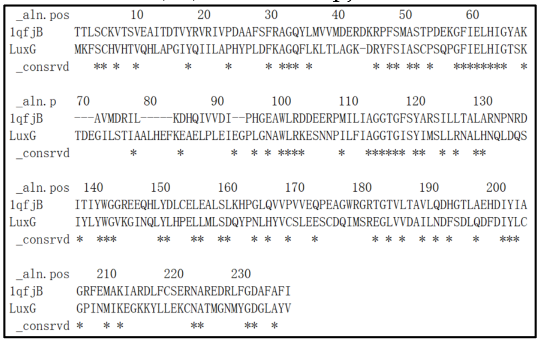

(38) LuxG *vs* 1qfj-B

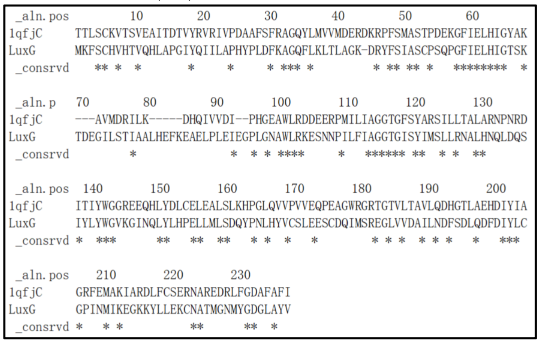

(39) LuxG *vs* 1qfj-C

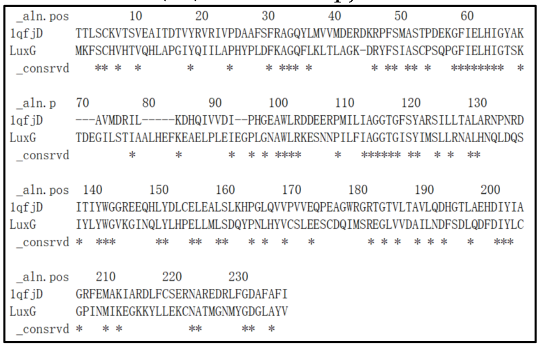

(40) LuxG *vs* 1qfj-D

Figure S1. The residue alignment between target protein and the template by MODELLER where the symbol "*" indicates that the corresponding residues are highly conserved.

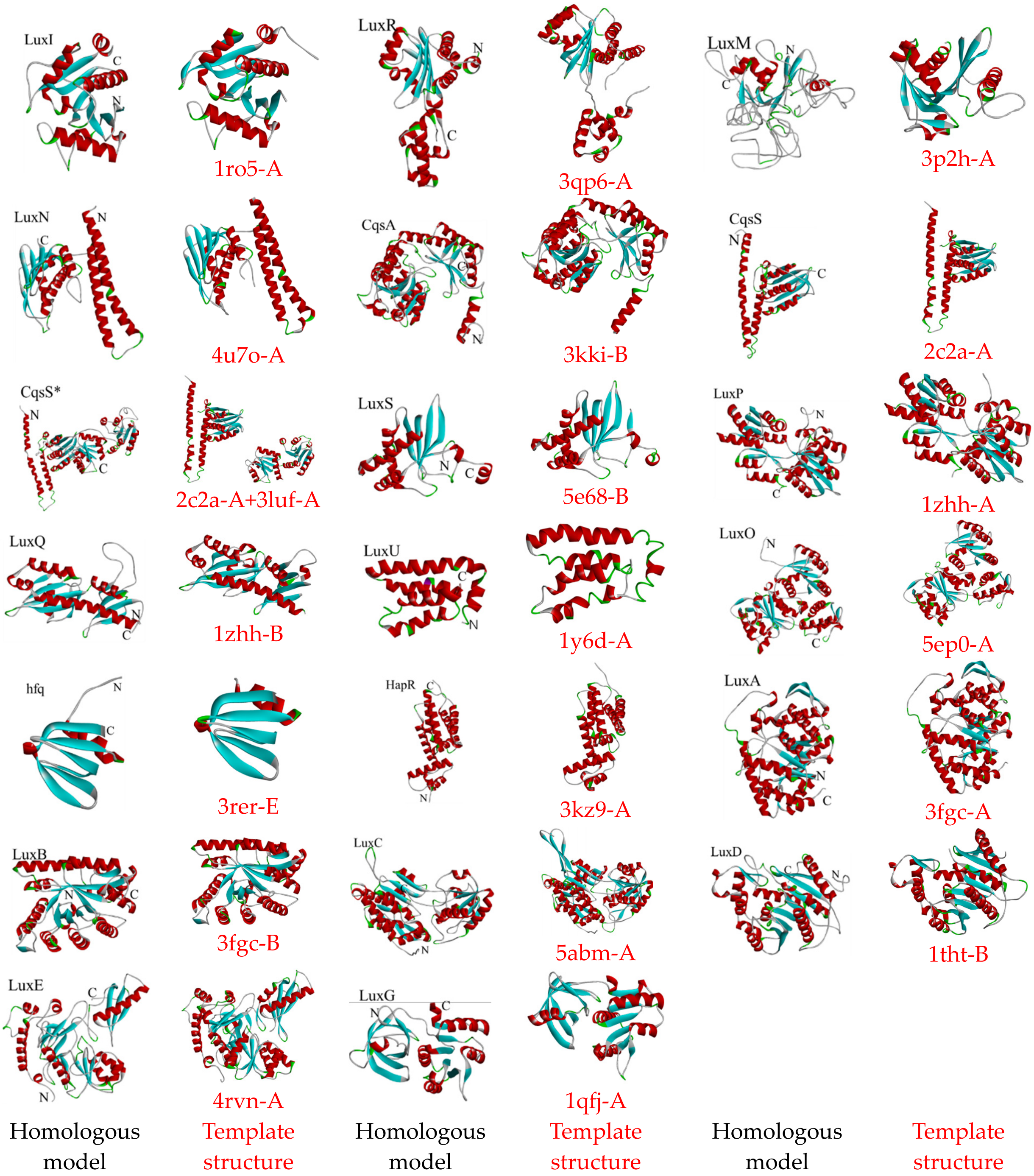

Figure S2. Ribbon diagram of protein and template monomers in the QS pathway of Q67 where loop, α-helices, and β-sheet are shown in silvery-white, red and green, respectively. "N" and "C" refer to the N-terminal and C-terminal of the protein monomer.

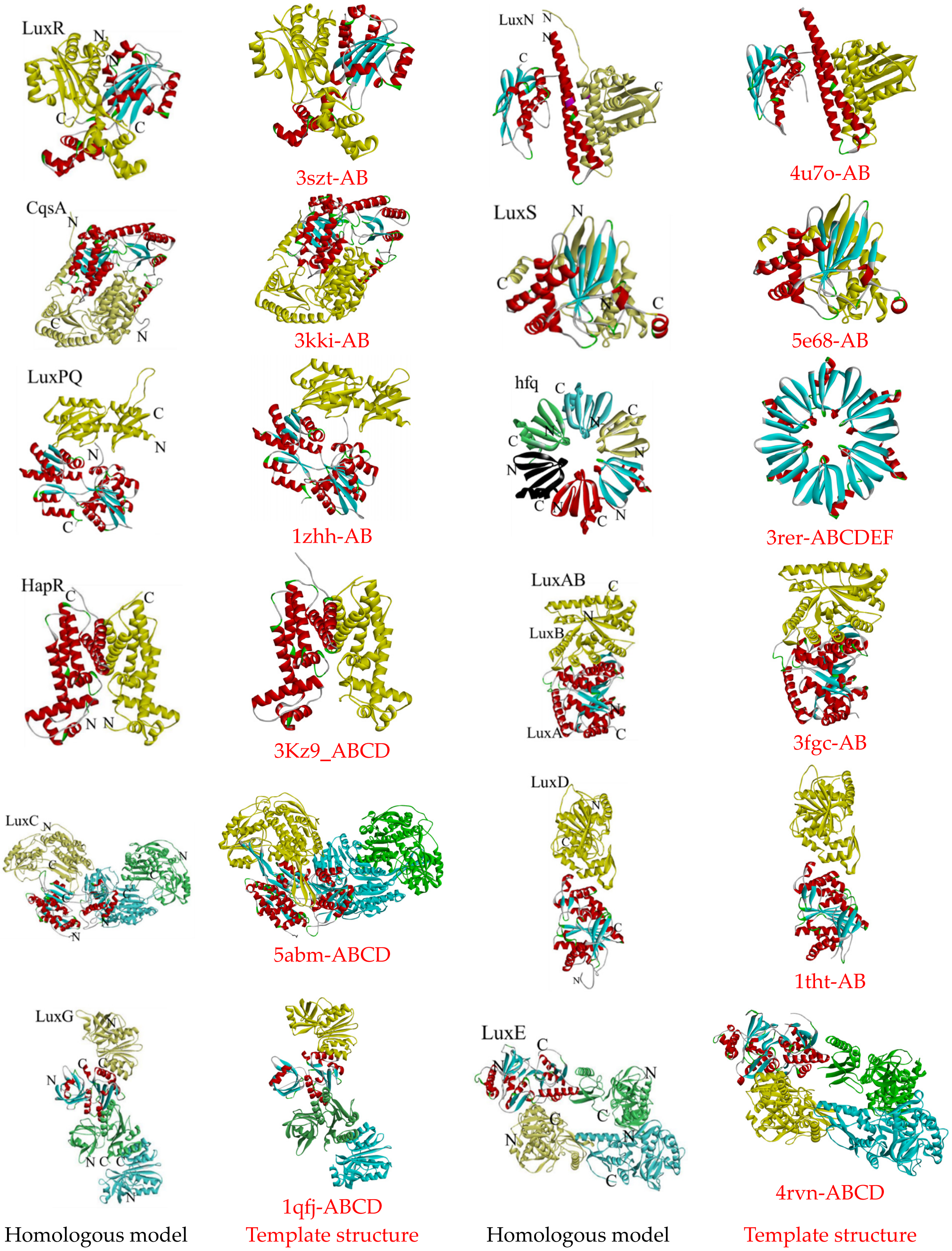

Figure S3. Ribbon diagram of protein and template oligomers in QS pathway of Q67 where colored ribbon diagram refers to chain A, yellow to chain B, blue to chain C, green to chain D, black to chain E, red to chain F. "N" and "C" refer to N-terminal and C-terminal of the protein oligomer.

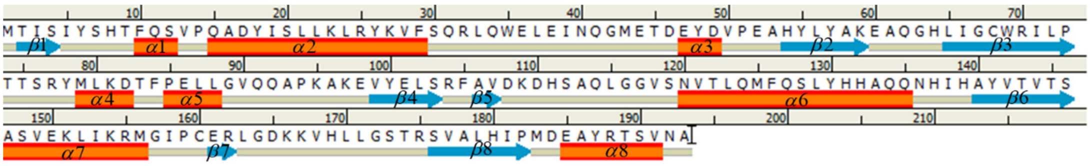

(a) LuxI_A

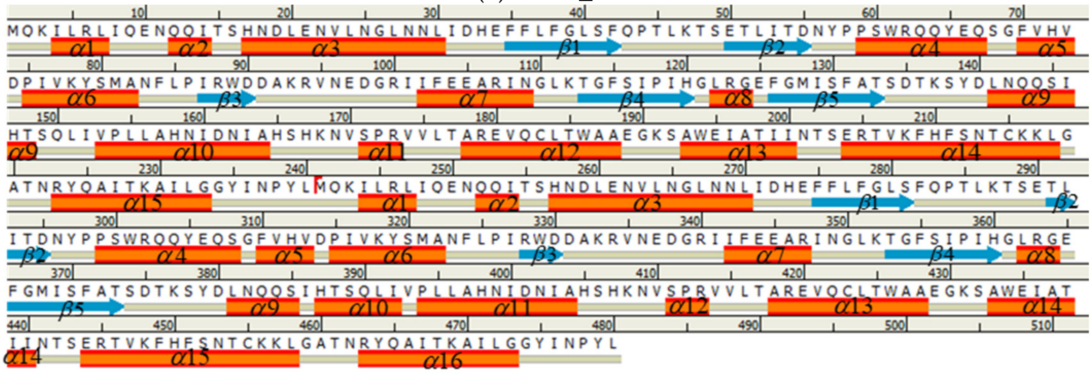

(b) LuxR_AB

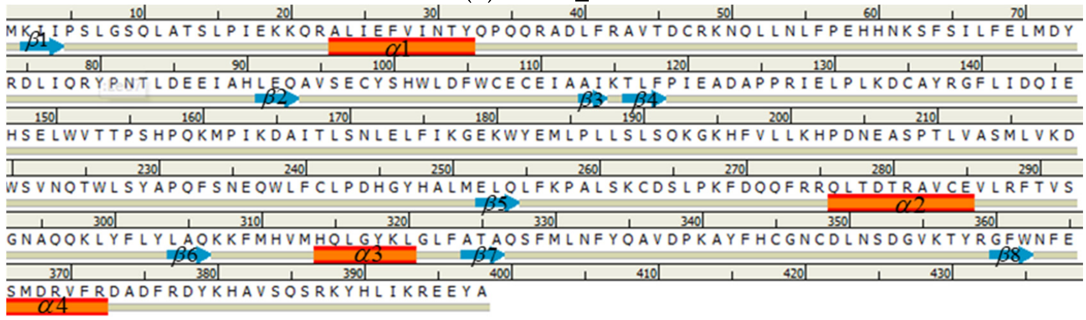

(c) LuxM_A

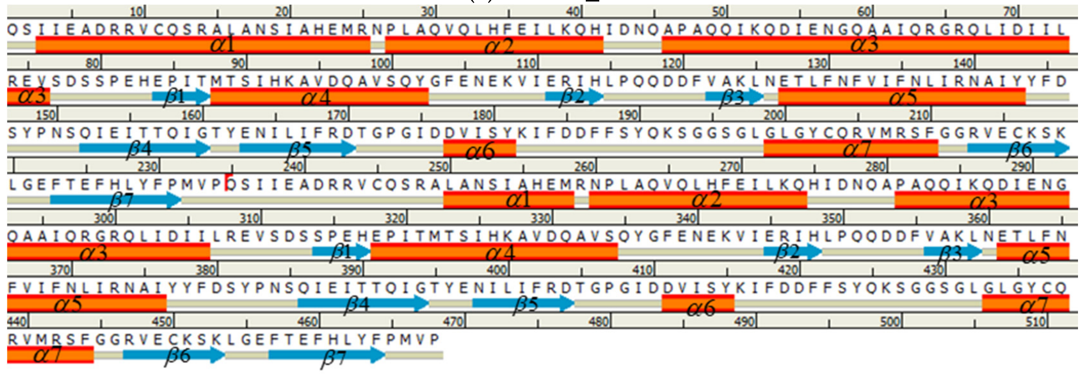

(d) LuxN_AB

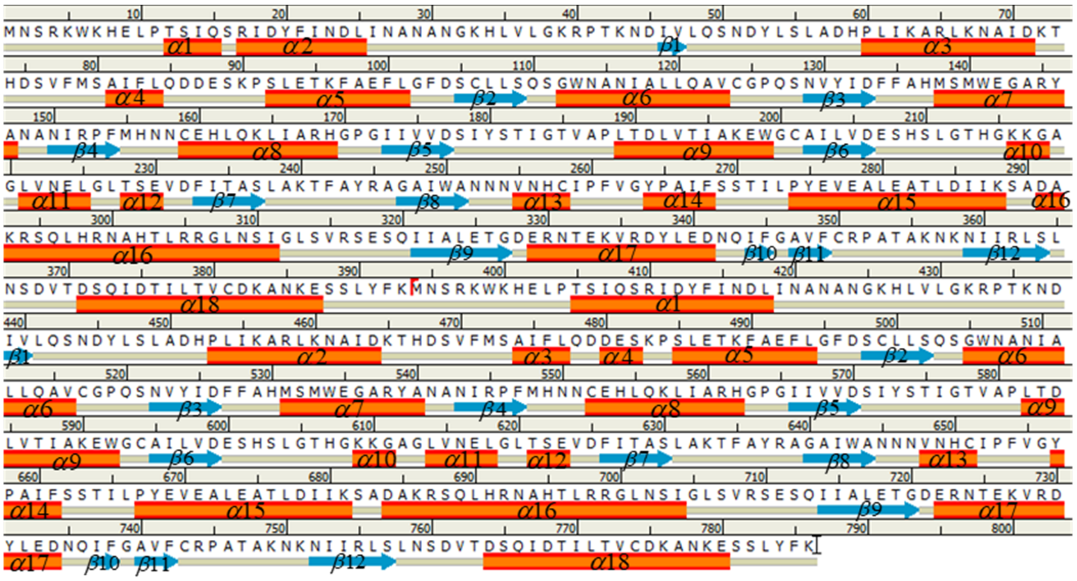

(e) CqsA_AB

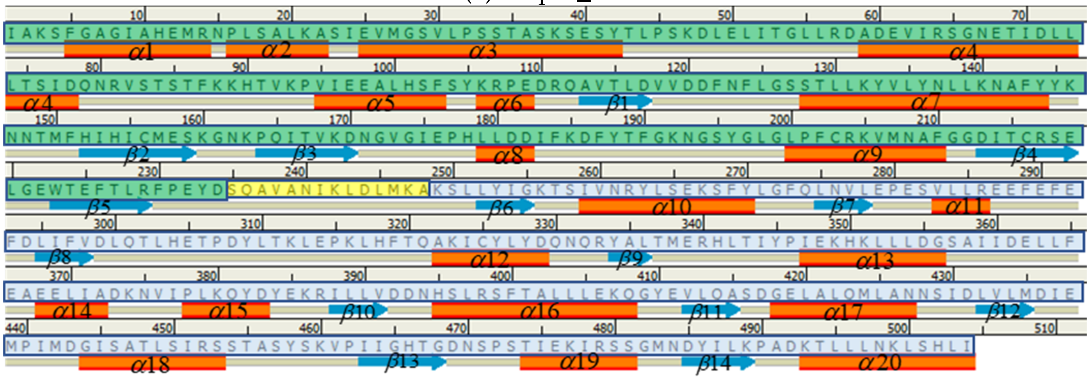

(f) CqsS_A

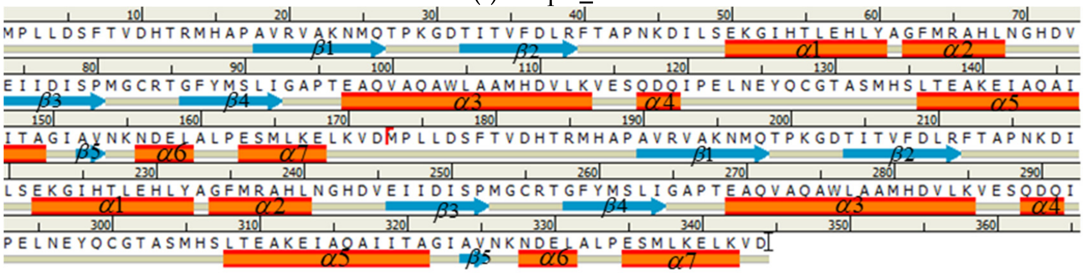

(g) LuxS_AB

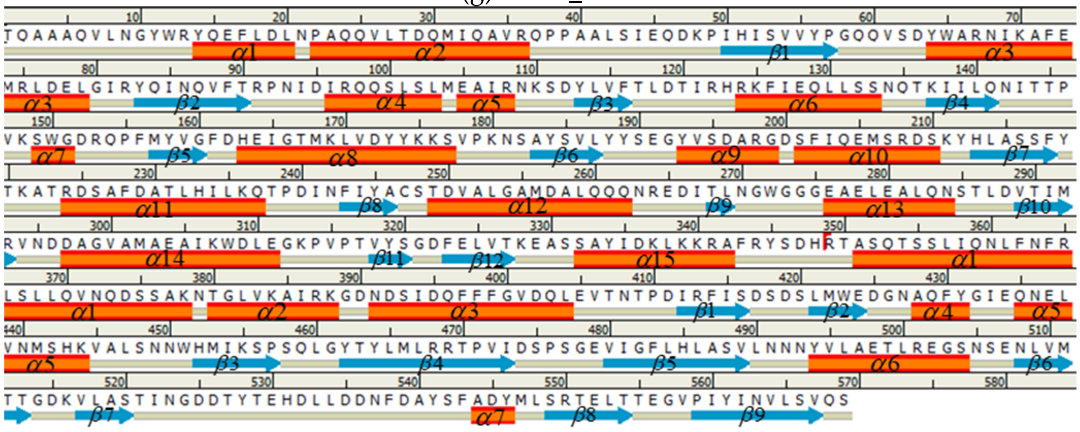

(h) LuxPQ

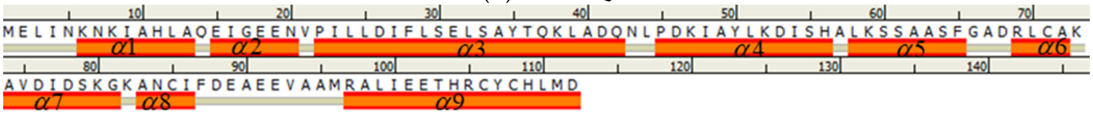

(i) LuxU_A

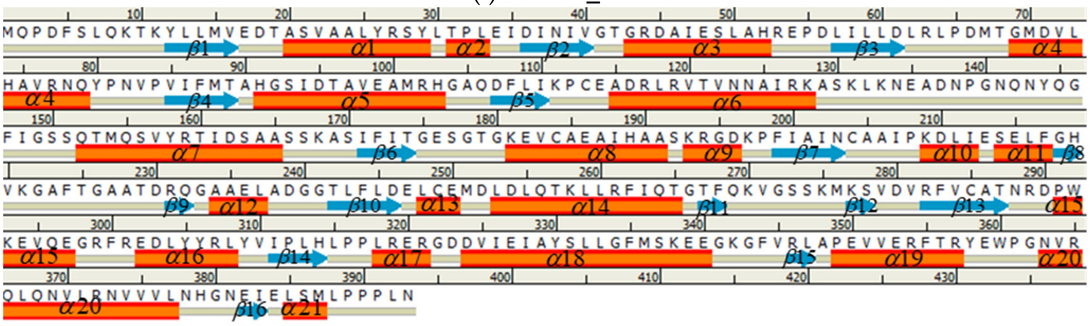

(j) LuxO_A

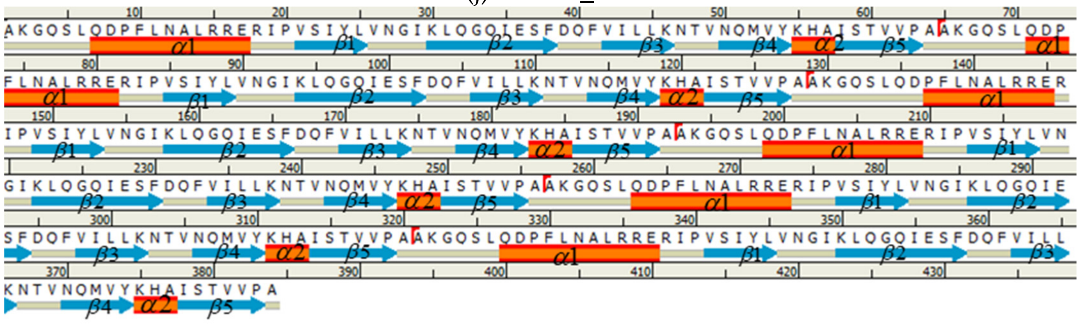

(k) Hfq_ABCDEF

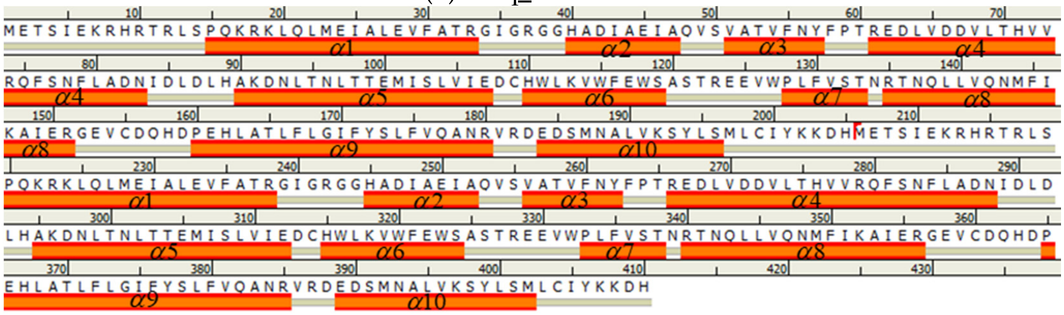

(l) HapR_AB

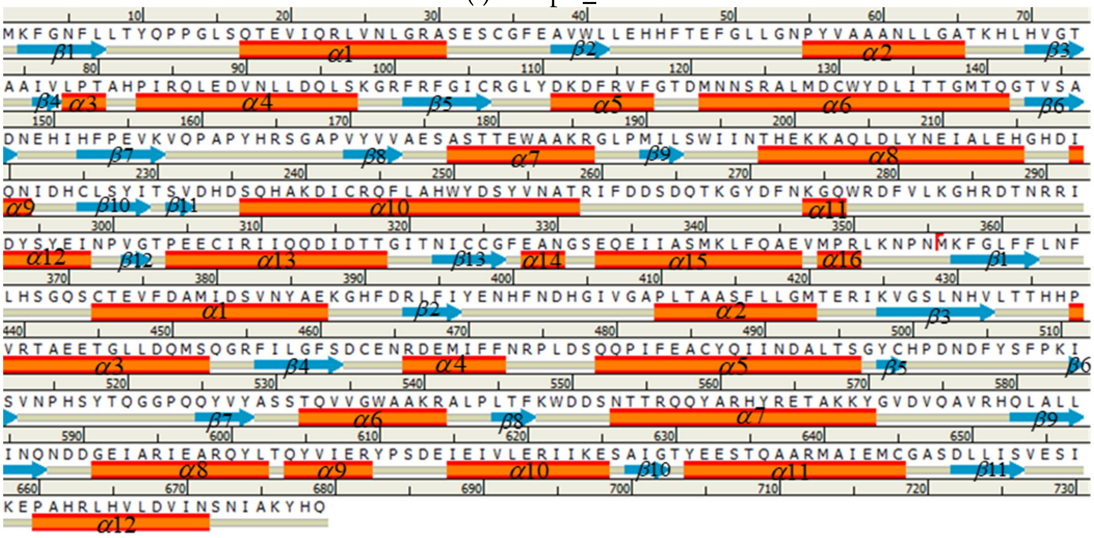

(m) LuxAB

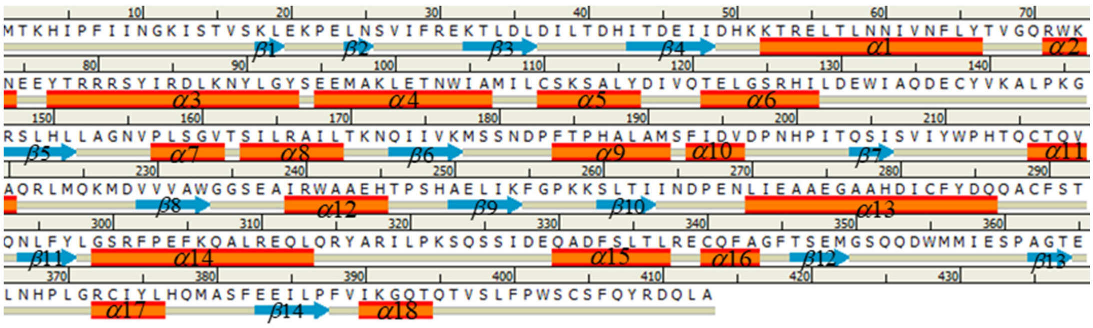

(n) LuxC_A

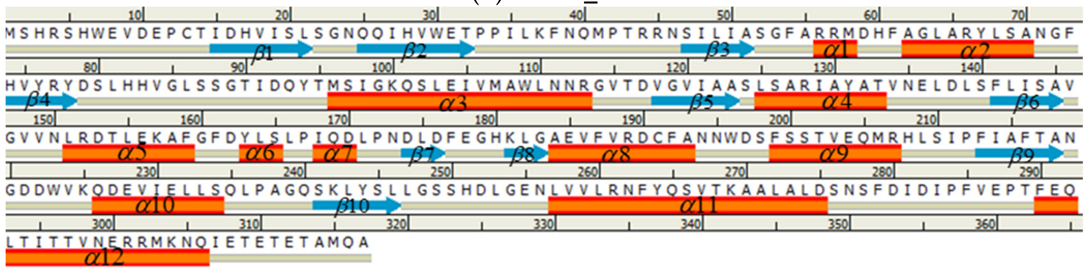

(o) LuxD_A

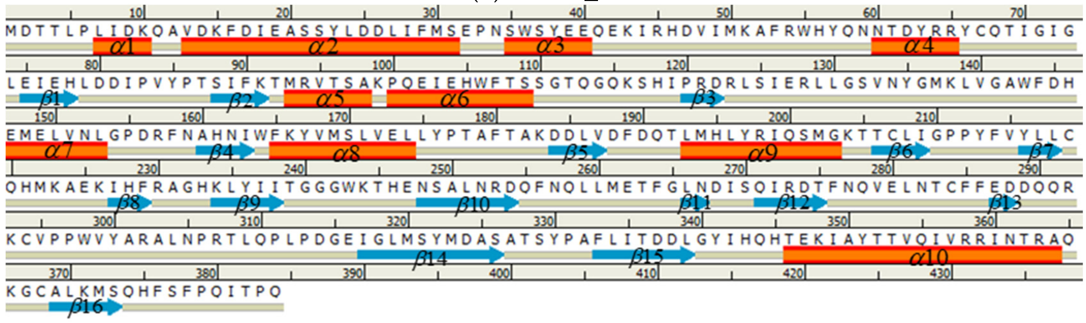

(p) LuxE_A

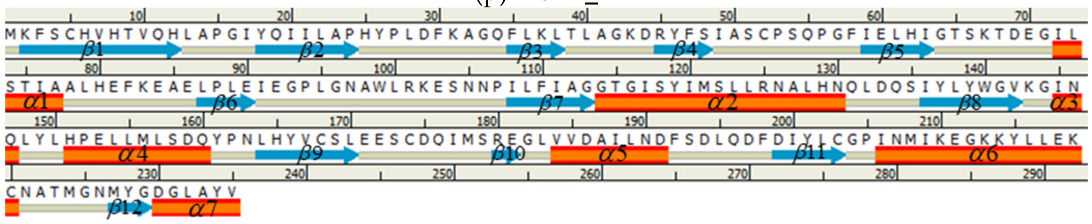

(q) LuxG_A

Figure S4. Sequence and topology diagram of 17 functional protein models where different chains are separated by symbols ‘ [illegible] ’. Blue arrows refer to numbered β-sheets from N-terminal to C-terminal and orange column to numbered α-helices from N-terminal to C-terminal. Since subunit chains do not have exactly the same secondary structures in the template proteins, the ABCDEF after the protein name represents the A to F chains of the protein respectively. Specially, for CqsS monomer with double template modeling, region based on template 2c2a is colored by green and region is based on template 3luf is colored by blue.

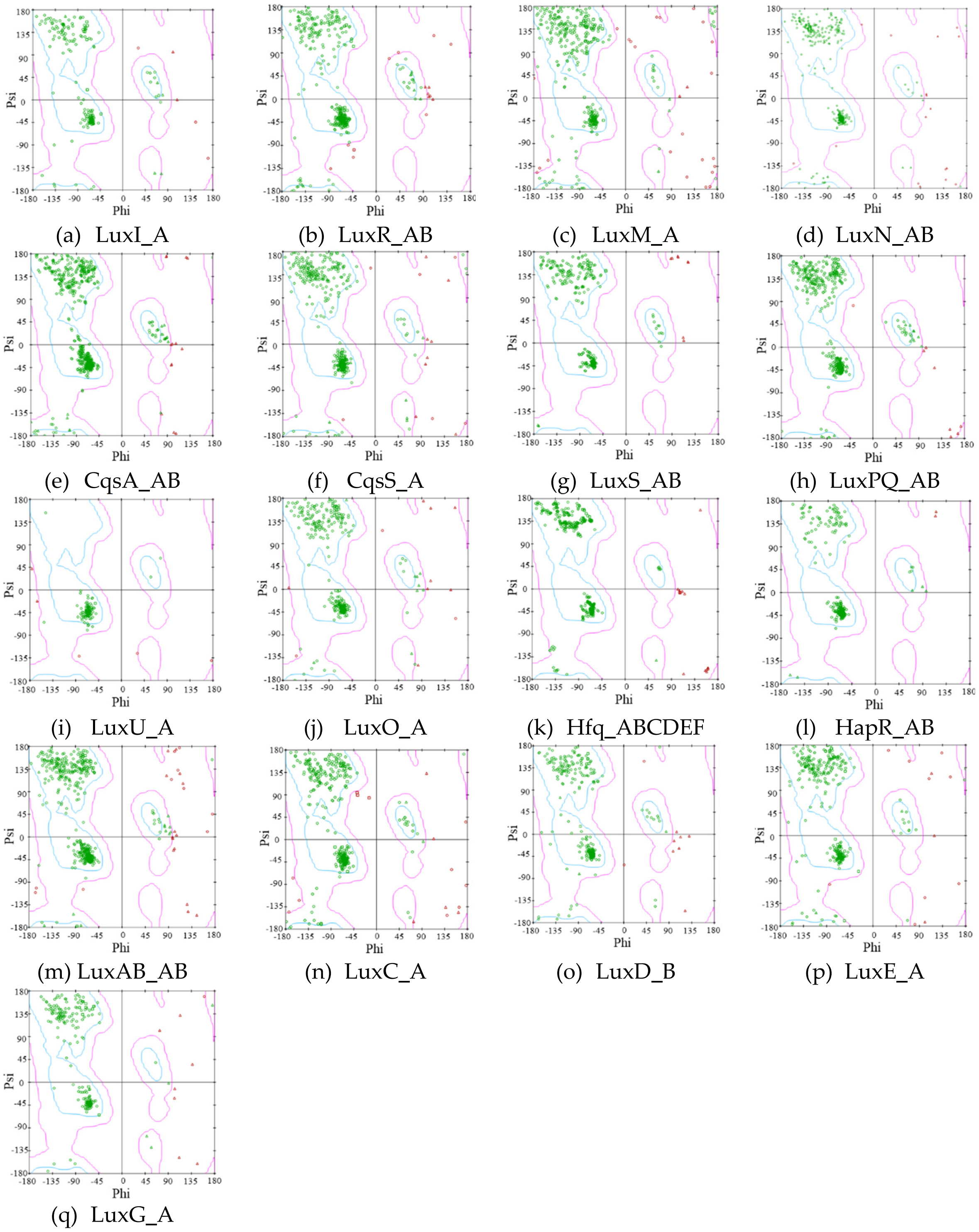

Figure S5. Ramachandran plot of 17 function proteins where green circles refer to residues in core regions (in blue closed line), green triangles to residues in additional allowed regions (in red closed line and outside blue closed line), red circles to residues in generously allowed regions (outside red closed line) and red triangles refer to residues in disallowed regions (outside red closed line).
